# Supplementary material for: Construction of emissive ruthenium(II) metallacycle over 1000 nm wavelength for in vivo biomedical applications
Source: Nat Commun. 2022 Apr 14;13:2009. doi: 10.1038/s41467-022-29572-2 (PMC9010459; doi:10.1038/s41467-022-29572-2)
Supplement: Supplementary file 1 — Supplementary Information [file 41467_2022_29572_MOESM1_ESM.pdf]

Supplementary Information

Construction of Emissive Ruthenium(II) Metallacycle over 1000 nm Wavelength for in vivo Biomedical Applications

Yuling Xu,[1,#] Chonglu Li,[1,#] Shuai Lu,[2,3] Zhizheng Wang,[1,4] Shuang Liu,[5] Xiujun Yu,[2,3] Xiaopeng Li,[2,3]* Yao Sun[1]*

[1]Key Laboratory of Pesticides and Chemical Biology, Ministry of Education, International Joint Research Center for Intelligent Biosensor Technology and Health, College of Chemistry, Central China Normal University, Wuhan, Hubei 430079, China.

[2]Shenzhen University General Hospital, Shenzhen University Clinical Medical Academy, Shenzhen, Guangdong 518055, China

[3]College of Chemistry and Environmental Engineering, Shenzhen University, Shenzhen, Guangdong 518060, China.

[4]Guangdong Provincial Key Laboratory of Luminescence from Molecular Aggregates, South China University of Technology, Guangzhou, Guangdong 510640, China.

[5]School of Materials Science and Engineering, Wuhan University of Technology, Wuhan, Hubei 430070, China.

[#]These authors contributed equally: Yuling Xu, Chonglu Li.

Table of contents

1. Supplementary Methods…………………………………………………………...…………...S3-4

2. Supplementary Tables…………………………….…………………………….……………………... S5

3. Supplementary Figures………………………….…………………….....…..……….……...S6-26

4. Synthetic Procedures and Characterization Data……….……………….…………………S27-45

5. Supplementary References…………………………………..……….….………………....S46

1. Supplementary Methods

Materials. Dulbecco's modified eagle medium (DMEM), Roswell Park Memorial Institute (RPMI) 1640, and fetal bovine serum (FBS) were purchased from Gibco (Australia). 5-Aminolevulinic acid (5-ALA), cisplatin, 3-(4, 5-dimethylthiazol-2-yl)-2, 5-diphenyltetrazolium bromide (MTT), trypsin–EDTA, Hoechst 33342, Annexin V-FITC apoptosis detection kit, crystal violet, caspase 3 activity assay kit, carbonyl cyanide m-chlorophenylhydrazone (CCCP), 2',7'-dichlorofluorescein diacetate (H_2-DCFH), 1,3-diphenylisobenzofuran (DPBF), and deoxyribonucleic acid sodium salt from calf thymus (ctDNA) were purchased from Sigma-Aldrich (Poole, UK). LysoTracker Green DND-26, Mito-Tracker Red CMXRos, propidium iodide (PI), acridine orange (AO), 5,5',6,6'-tetrachloro-1,1',3,3'-tetraethylbenzimidazolylcarbocyanine iodide (JC-1), RNase A, and 2,2,6,6-tetramethylpiperidine (TEMP) were purchased from Thermo Fisher Scientific (USA). Transwell inserts with 8.0 μm-pore size filters were purchased from Corning (USA). 1,2-distearoyl-sn-glycero-3-phosphoethanolamine-N-[methoxy(polyethyleneglycol)-5000] (DSPE-PEG5000) was purchased from Avanti Polar Lipids.

Instruments. NMR spectra were obtained using a Varian Inova 400 MHz NMR spectrometer. Mass spectra were recorded on a Micromass Quattro II triple-quadrupole mass spectrometer or Synapt G2-Si mass spectrometer using electrospray ionization with a MassLynx operating system (Waters, USA). Absorption spectra were measured on a UV−vis-NIR spectrophotometer (Shimadzu UV-3600, Japan). Fluorescence spectra were measured on a Fluorolog-3 spectrofluorometer (Horiba Jobin Yvon, France) and the fluorescence emitting within the NIR-II region were measured by a Cary Eclipse fluorophotometer. The laser of 808-nm wavelength was purchased from Beijing Hi-Tech Optoelectronic (China). Cellular NIR-II fluorescence images were captured by NIR-II fluorescence

microscope (Suzhou NIR-Optics, China). Other cellular images were captured on invert fluorescence microscope (Zeiss Fluorescence Microscope, Germany). *In vivo* NIR-II imaging was carried on Suzhou NIR-Optics imaging system. ESR measurements were performed on a JES-FA200 spectrometer (JEOL, Japan). Photothermal temperature and images were monitored by an infrared thermal imaging camera (Fluke, Ti400, USA). The inductively coupled plasma mass spectrometer (ICP-MS) analysis was conducted on a quadrupole ICP-MS (7700x ICP-MS system, Agilent Technologies, USA). Laser ablation inductively coupled plasma mass spectrometry (LA-ICP-MS) was conducted using an NWR image laser ablation system (ESI, USA) with a 266 nm Nd: YAG laser and a high performance two volume ablation chamber (TwoVol2) for laser ablation, and a quadrupole ICP-MS (PerkinElmer, NexION 2000D, USA) coupled with an NWR image laser ablation system for element measurement. Flow cytometry was performed on Fortessa X20 (BD Biosciences, USA). Hydrodynamic diameter and zeta potential were tested by Malvern Zetasizer Nano ZS. Transmission electron microscopy (TEM) images were captured on Hitachi HT-7700 (Japan).

2. Supplementary Tables

Supplementary Table 1. Photophysical properties of Ru1085.

Solvent	λ_{abs}(nm)[a]	ε_{max}(×10^{4} M^{-1} cm^{-1})[b]	λ_{em}(nm)[c]	Φ_f(%)[d]
Dichloromethane (DCM)	874	4.67	1085	0.084
Tetrahydrofuran (THF)	837	4.36	1045	0.052
Acetonitrile (ACN)	869	4.11	1120	0.026
Dimethyl sulfoxide (DMSO)	916	4.37	1122	0.038
N, N-Dimethylformamide (DMF)	898	4.21	1115	0.019

a. Maximum absorption peak of **Ru1085** in different solvents; b. Molar extinction coefficient of **Ru1085** in different solvents; c. Maximum emission peak of **Ru1085** in different solvents; d. Quantum yield of **Ru1085** in different solvents.

3. Supplementary Figures

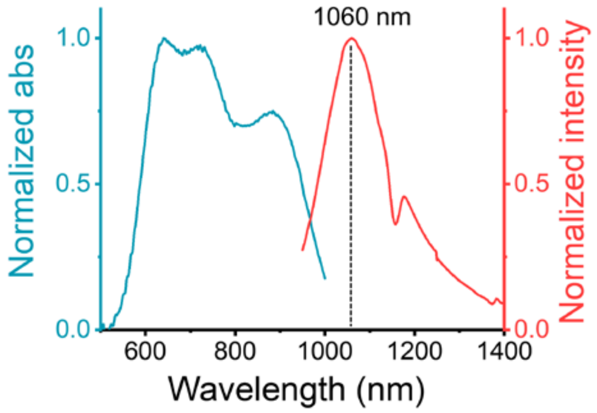

Supplementary Figure 1. Normalized absorption and normalized fluorescence emission spectra of **1** in DCM.

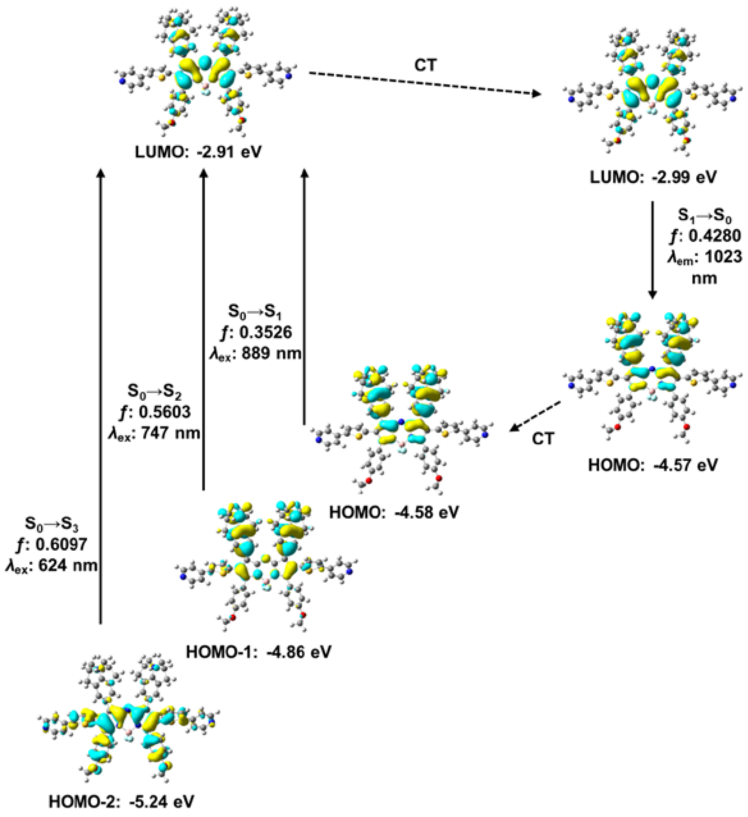

Supplementary Figure 2. The theoretical calculation of **1** at the B3LYP/6-31G(d) level using Gaussian 09. Rationalization of the UV−vis absorption and emission of ligand **1**: the geometry relaxation and the frontier molecular orbitals involved in the excitation (the left three columns) and emission (the right column) of **1**. CT stands for conformation transformation.

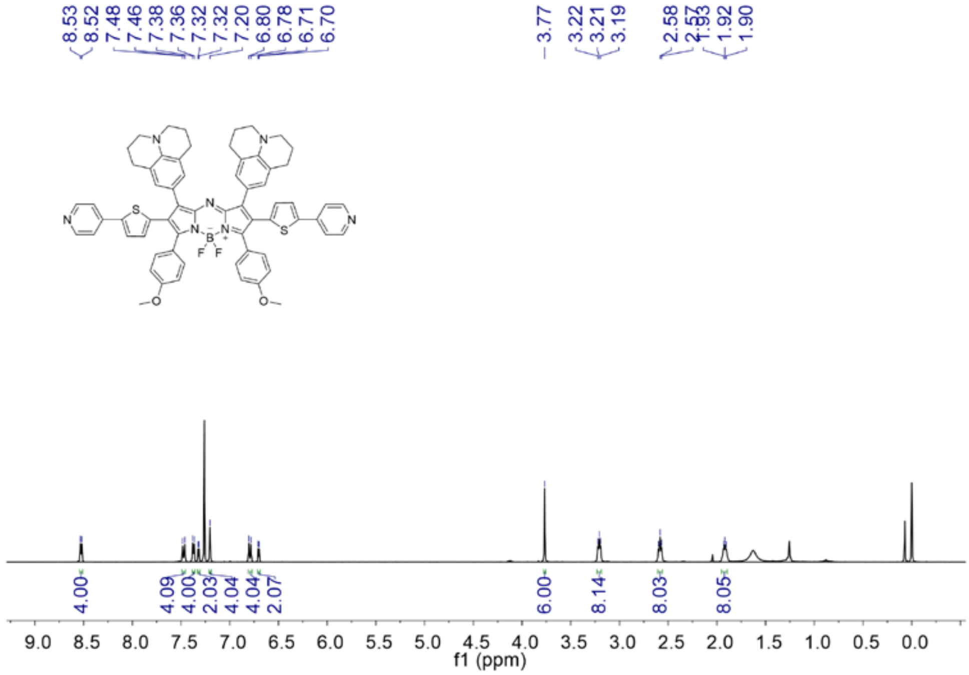

Supplementary Figure 3. ^{1}H NMR spectrum of ligand **1**.

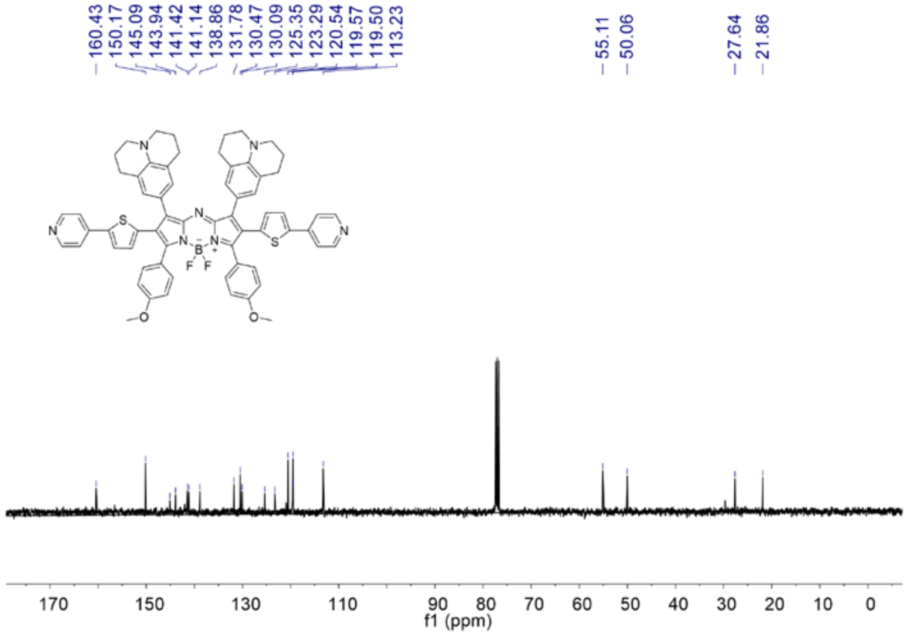

Supplementary Figure 4. ^{13}C NMR spectrum of ligand **1**.

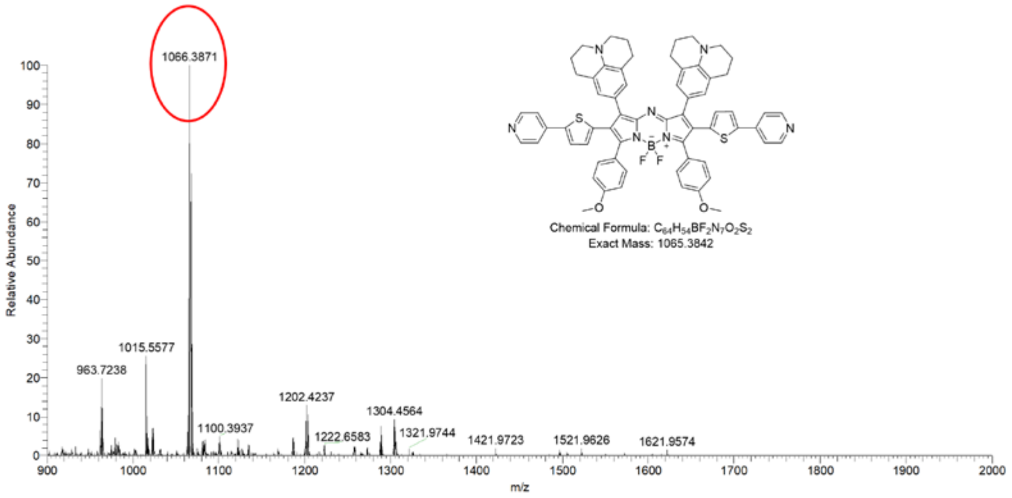

Supplementary Figure 5. ESI-MS spectrum of ligand **1**.

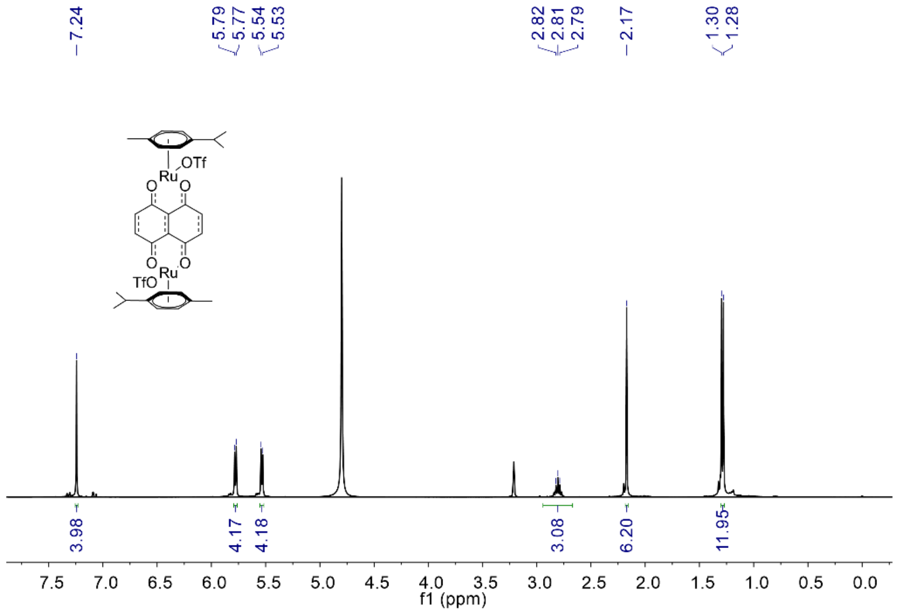

Supplementary Figure 6. ^{1}H NMR spectrum of **2**.

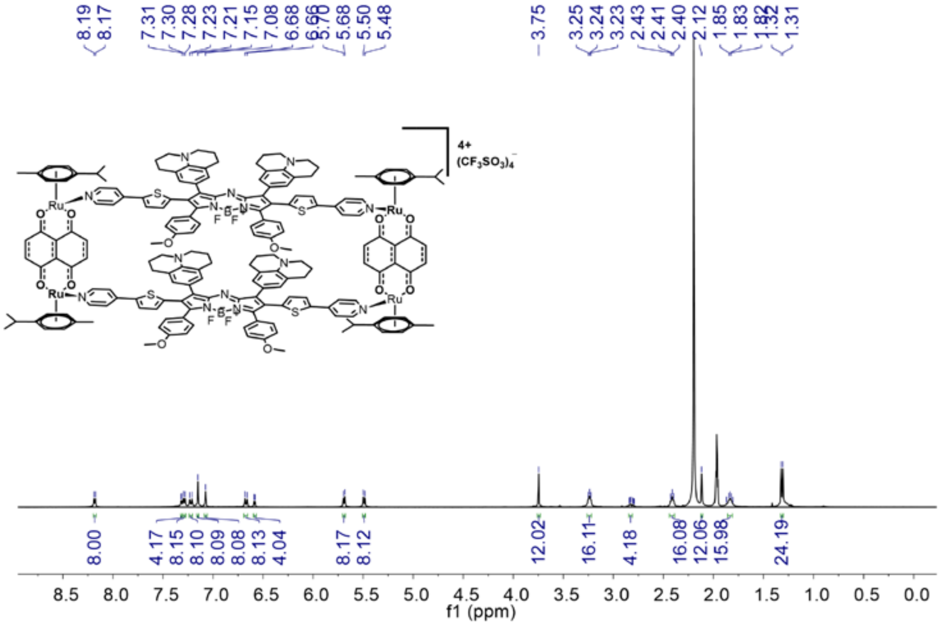

Supplementary Figure 7. ^{1}H NMR spectrum of **Ru1085**.

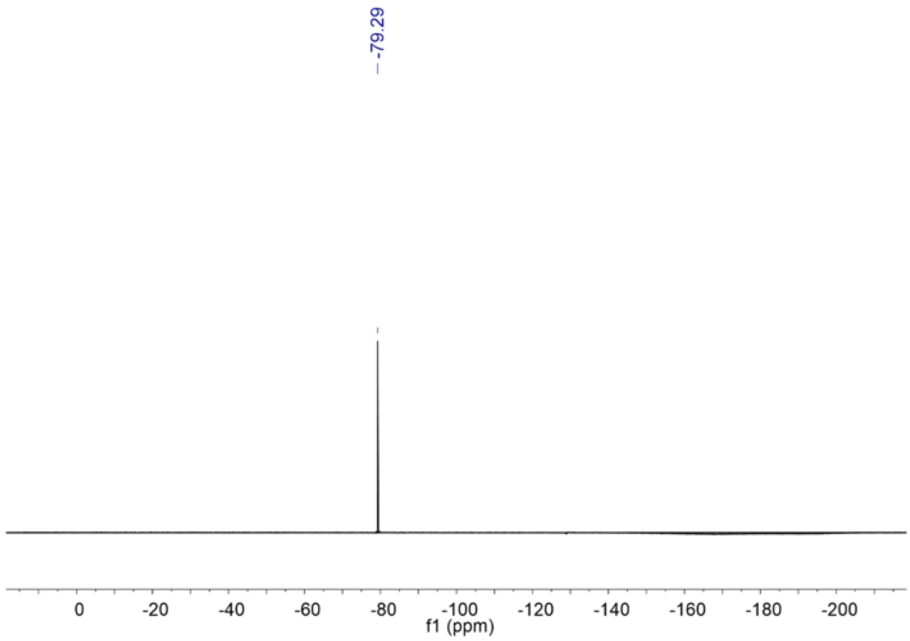

Supplementary Figure 8. ^{19}F NMR spectrum of **Ru1085**.

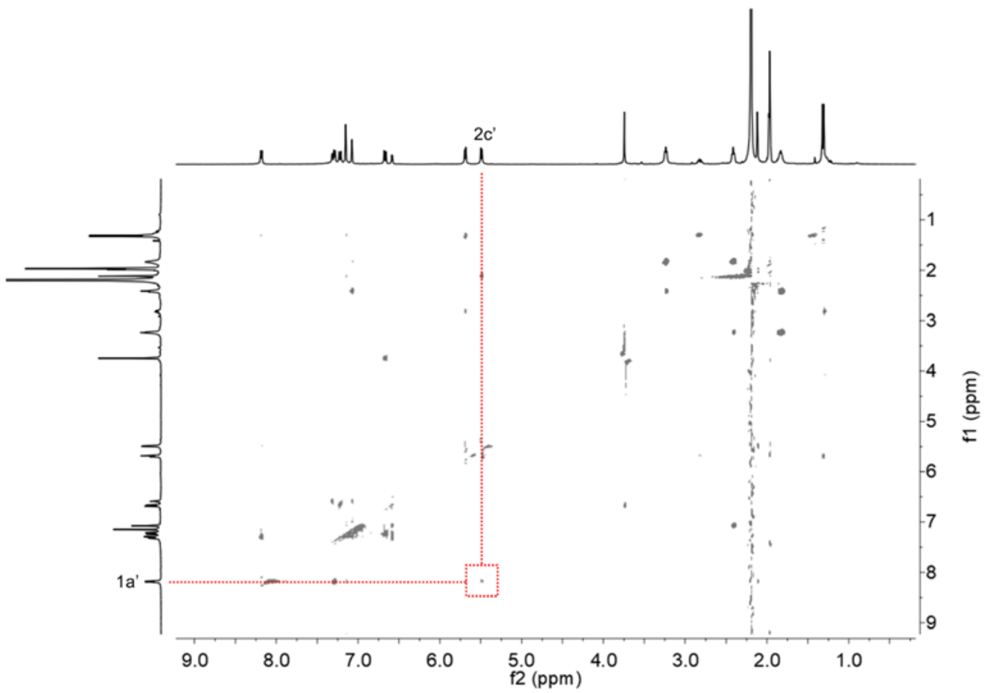

Supplementary Figure 9. 2D ^{1}H-^{1}H ROESY spectrum of **Ru1085**.

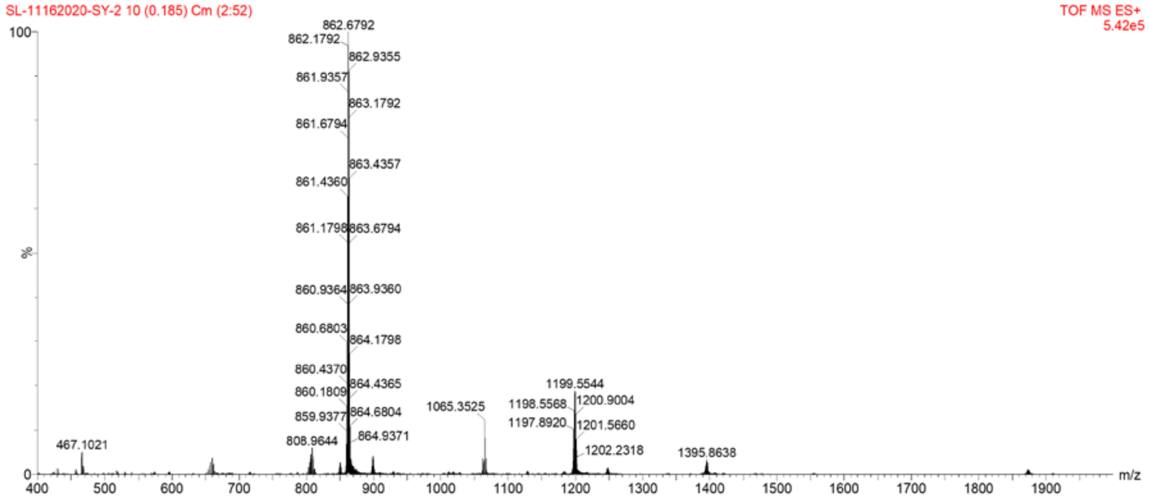

Supplementary Figure 10. ESI-TOF-MS spectrum of **Ru1085**.

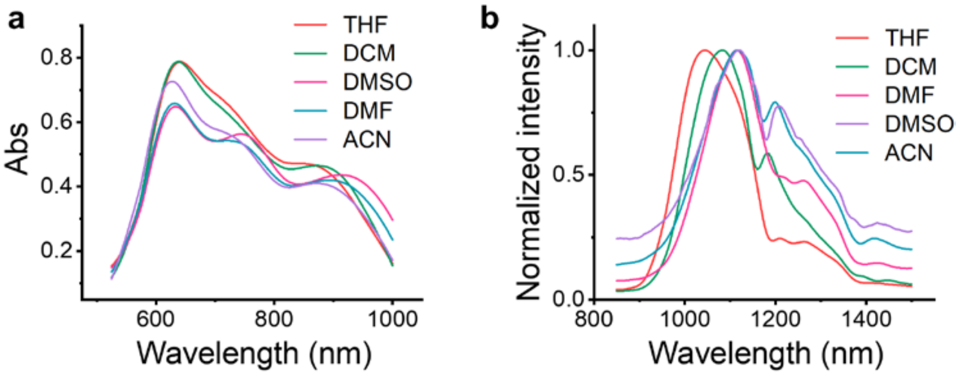

Supplementary Figure 11. Absorption (a) and normalized fluorescence emission (b) spectra of **Ru1085** in tetrahydrofuran (THF), dichloromethane (DCM), dimethyl sulfoxide (DMSO), N, N-dimethylformamide (DMF) and acetonitrile (ACN).

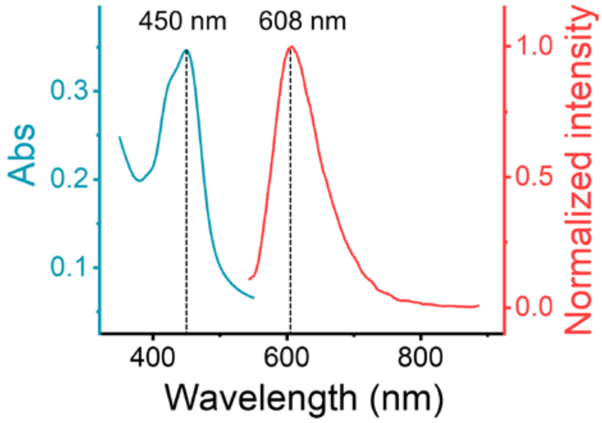

Supplementary Figure 12. Absorption and normalized fluorescence emission spectra of $Ru(bpy)_3Cl_2$ in acetonitrile.

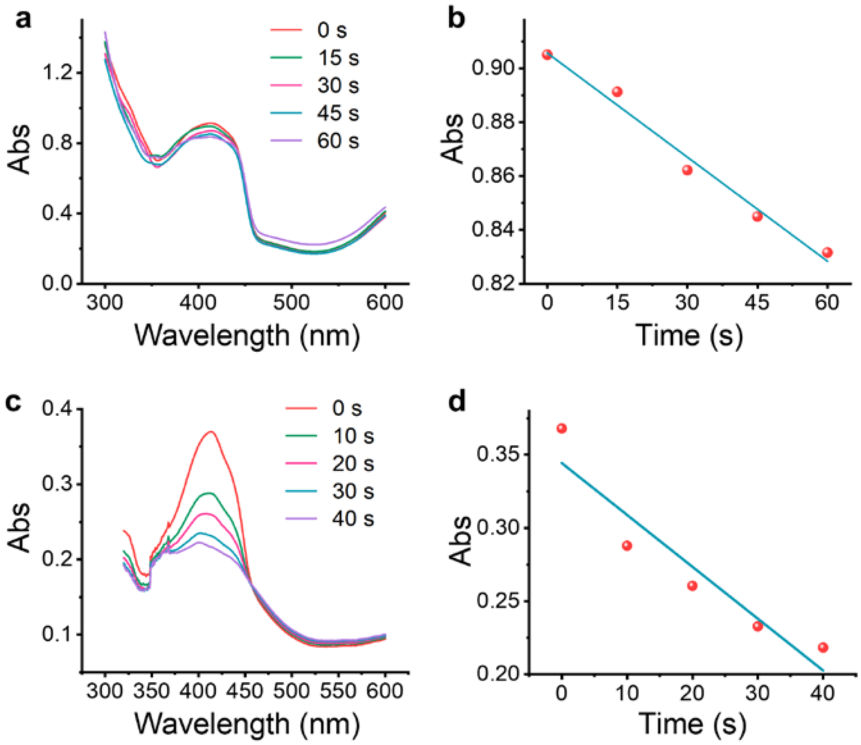

Supplementary Figure 13. $^{1}O_2$ generation of **Ru1085** (20 μM) (a) and ICG (20 μM) (c) using DPBF as a probe with 808 nm laser illumination (0.8 W cm^{-2}) for various time. Linear calibration curve for the absorbance of DPBF plus **Ru1085** (b) or ICG (d) to illumination time.

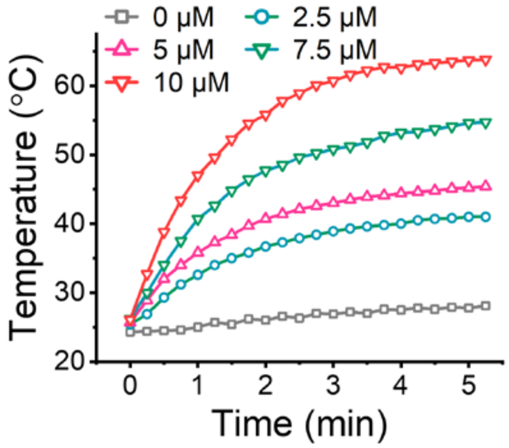

Supplementary Figure 14. Photothermal curves of **Ru1085** with 808 nm laser illumination (0.8 W cm^{-2}) for 5 min at various concentration.

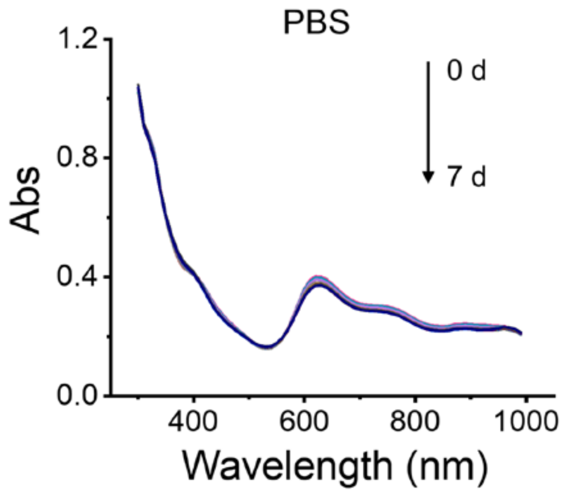

Supplementary Figure 15. Absorption spectra of **Ru1085** (50 μM) incubated in PBS buffer and stored for various time.

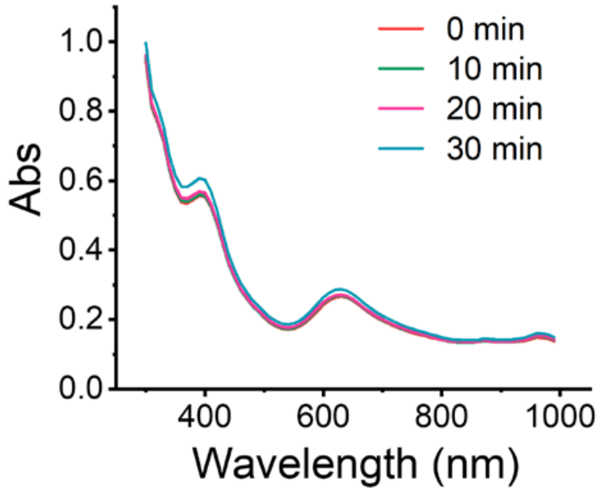

Supplementary Figure 16. Absorption spectra of **Ru1085** (50 μM) with 808 nm laser illumination (0.4 W cm^{-2}) for various time.

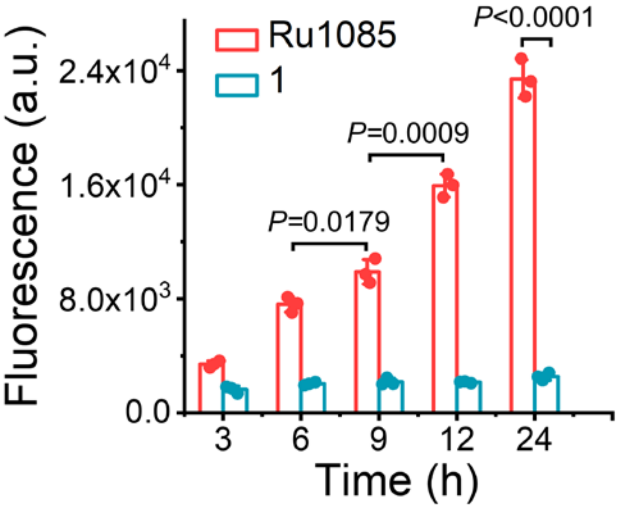

Supplementary Figure 17. Semi-quantitative analysis of Fig. 4a. Data are presented as mean ± s.d. (*n* = 3). Statistical differences were analyzed by Student's two-sided *t*-test. Source data are provided as a Source Data file.

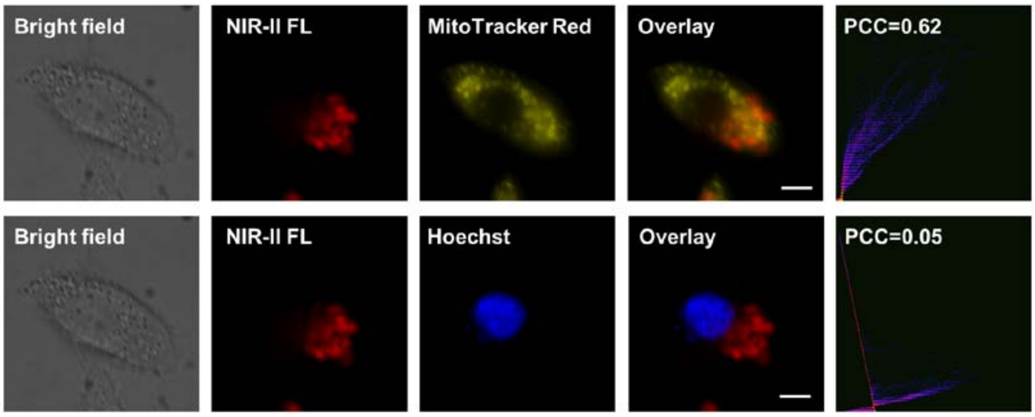

Supplementary Figure 18. Co-localization assay of **Ru1085** (10 µM) incubated with A549 cells for 6 h using Mito-Tracker® Red CMXRos as mitochondrial dye and Hoechst 33342 as nuclear dye. The Pearson correlation coefficients (PCC) were analyzed by ImageJ. Scale bars, 5 µm.

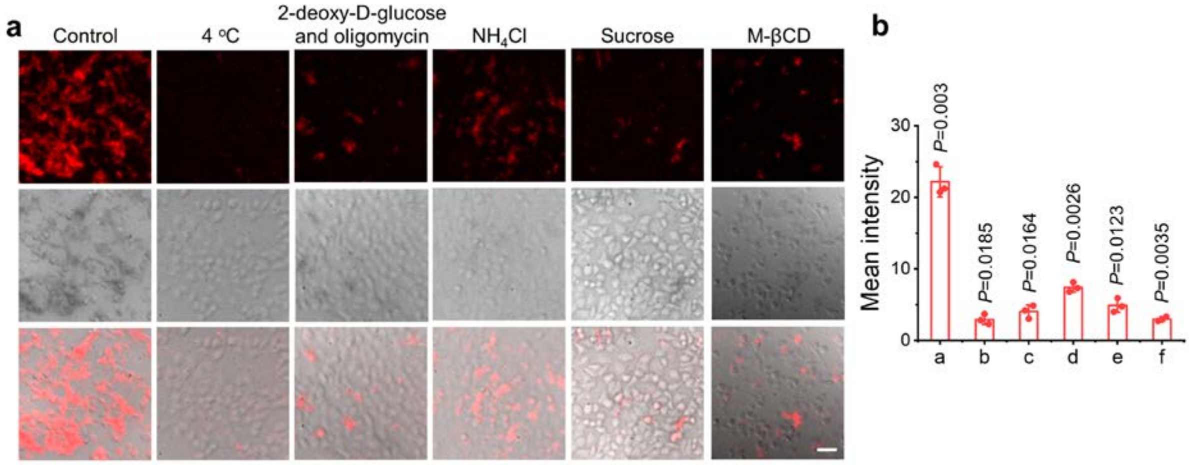

Supplementary Figure 19. Cell uptake mechanism study of **Ru1085** (10 μM) incubated with various inhibitors/conditions (control: incubation at 37 °C; 4 °C: incubation at 4 °C; 2-Deoxy-D-glucose with concentration at 50 mM and oligomycin with concentration at 5 μM for metabolic inhibition; NH_4Cl with concentration at 50 mM for endocytic inhibition; sucrose with concentration at 5 μM for clathrin mediated endocytic inhibition; M-βCD with concentration at 10 mM for caveolae mediated endocytic inhabitation). Scale bar, 50 μm. Data are presented as mean ± s.d. (n = 3). Statistical differences were analyzed by Student's one-sided t-test. Source data are provided as a Source Data file.

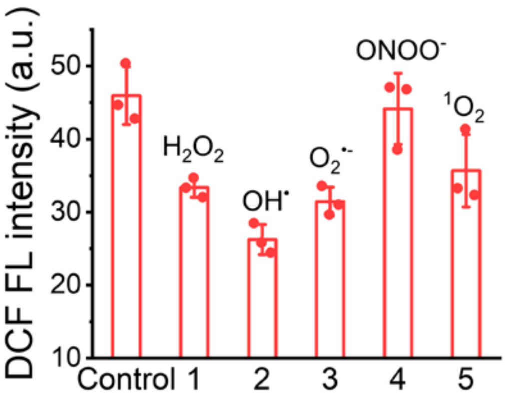

Supplementary Figure 20. Cellular ROS level in the presence of different ROS quenchers (1: sodium pyruvate, 2: D-mannitol, 3: tiron, 4: ebselen, 5: sodium azide). H_2-DCFH was used as ROS indicator. Data are presented as mean ± s.d. (n = 3). Source data are provided as a Source Data file.

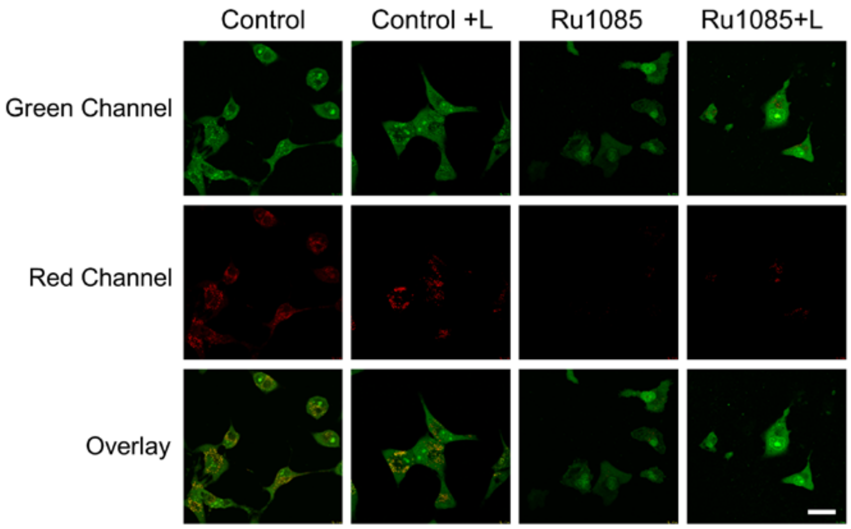

Supplementary Figure 21. After incubated with **Ru1085** (10 μM) or serum-free medium (control) and treated with or without 808 nm laser illumination (0.8 W cm^{-2}, 5 min), the A549 cells were taken AO staining. Scale bar, 20 μm.

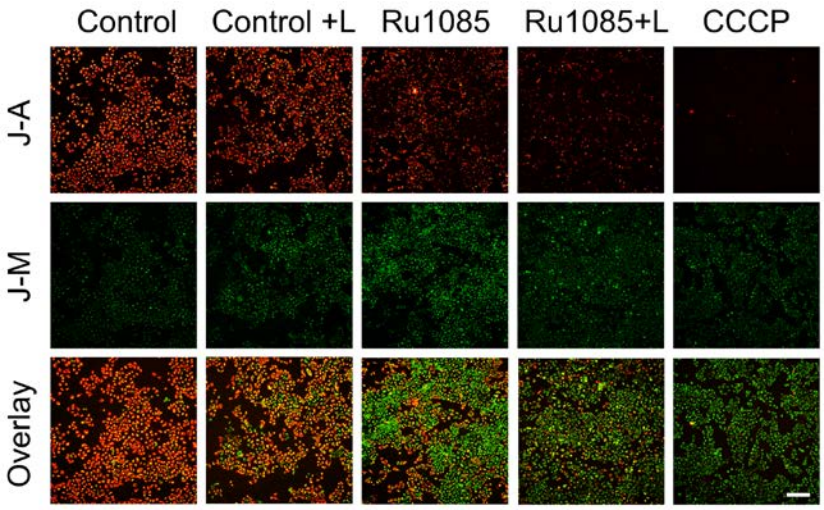

Supplementary Figure 22. After incubated with **Ru1085** (10 μM) or serum-free medium (control) and treated with or without 808 nm laser illumination (0.8 W cm^{-2}, 5 min), the A549 cells were taken JC-1 staining. Carbonyl cyanide m-chlorophenylhydrazone (CCCP) as positive control. Scale bar, 300 μm.

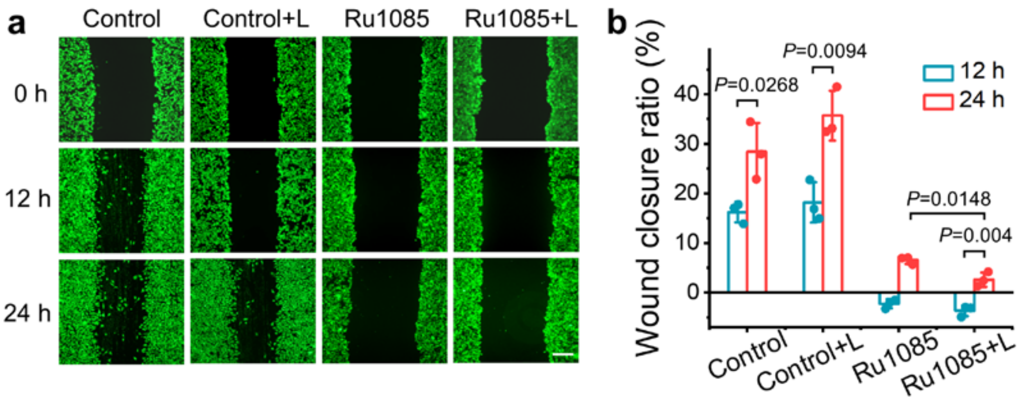

Supplementary Figure 23. Anti-migration capability of **Ru1085** by wound healing assay. (a) After incubated with **Ru1085** (10 μM) or serum-free medium (control) and treated with or without 808 nm laser illumination (0.8 W cm^{-2}, 5 min), A549 cells were imaged with calcein-AM staining. Scale bar, 300 μm. (b) The wound area was analyzed using the ImageJ software and the wound closure ratio was calculated. Data are presented as mean ± s.d. (n = 3). Statistical differences were analyzed by Student's two-sided t-test. Source data are provided as a Source Data file.

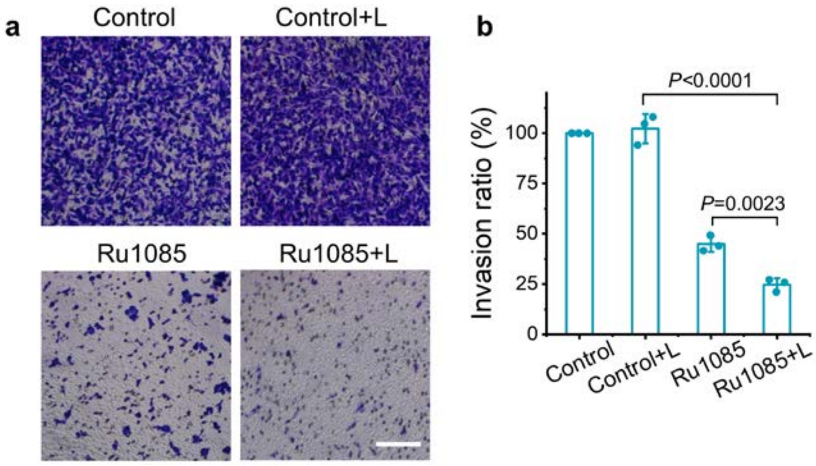

Supplementary Figure 24. Anti-invasion capability of **Ru1085** by transwell invasion assay. (a) After incubated with **Ru1085** (10 μM) or serum-free medium (control) and treated with or without 808 nm laser illumination (0.8 W cm^{-2}, 5 min), the A549 cells were taken crystal violet staining. Scale bar, 200 μm. (b) Invasion ratio was calculated by the optical density of crystal violet. Data are presented as mean ± s.d. (n = 3). Statistical differences were analyzed by Student's two-sided t-test. Source data are provided as a Source Data file.

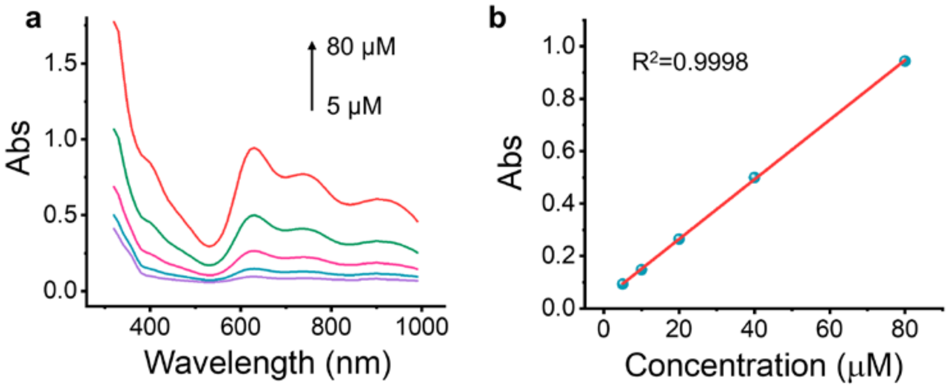

Supplementary Figure 25. (a) Absorption spectra of **Ru1085** at various concentration. (b) Plot of the absorbance as a function of the concentration of **Ru1085**. The encapsulation efficiency was calculated to be 31%.

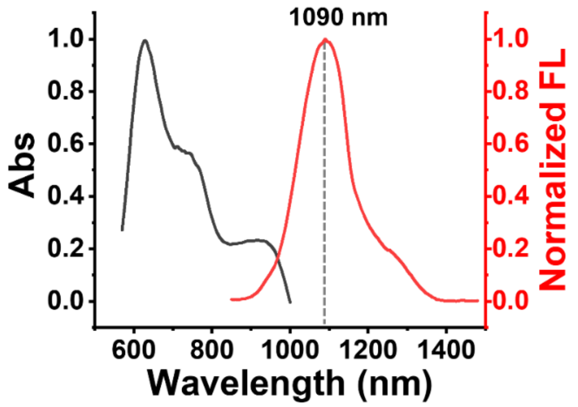

Supplementary Figure 26. Absorption and normalized emission spectra (λ_{ex} = 808 nm) of **Ru1085** NPs in water.

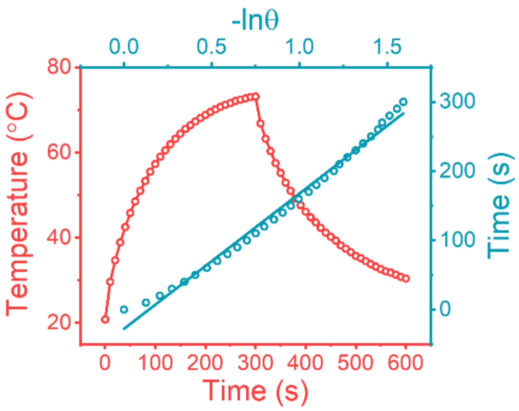

Supplementary Figure 27. Monitored temperature profile (red line) of **Ru1085** NPs illuminated for 300 s and followed by natural cooling, and linear time data versus –lnθ (blue line) from the cooling period.

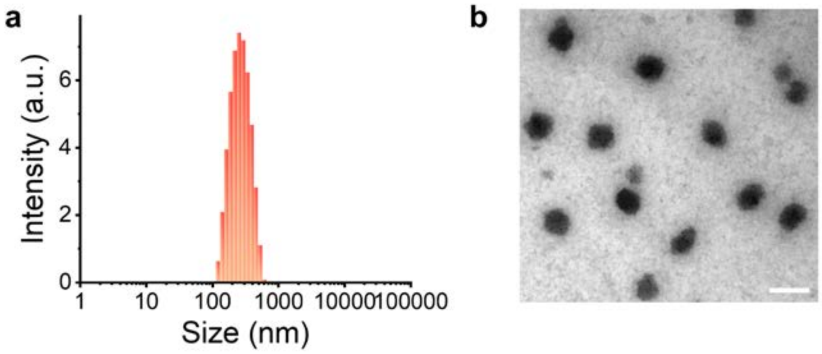

Supplementary Figure 28. DLS data (a) and TEM image (b) of **Ru1085** NPs. Scale bar, 300 nm.

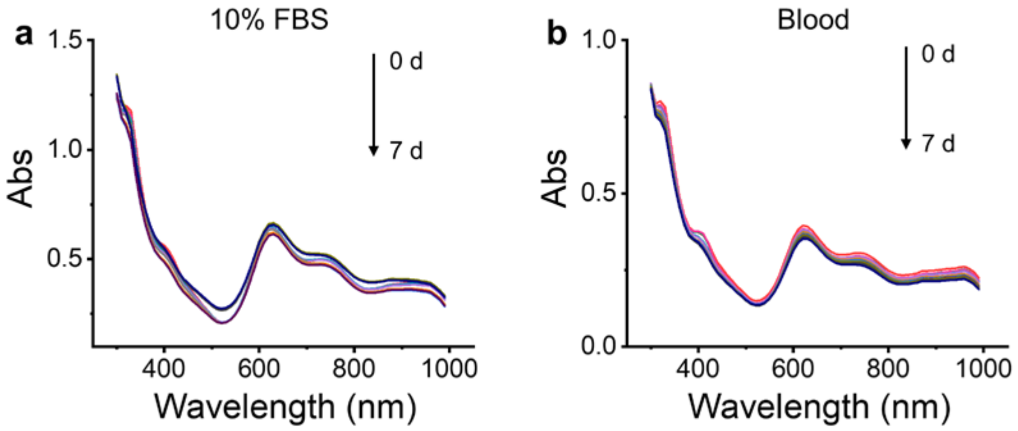

Supplementary Figure 29. Absorption spectra of **Ru1085** NPs (50 μM) incubated in 10% FBS (a) and whole blood (b) and stored for various time.

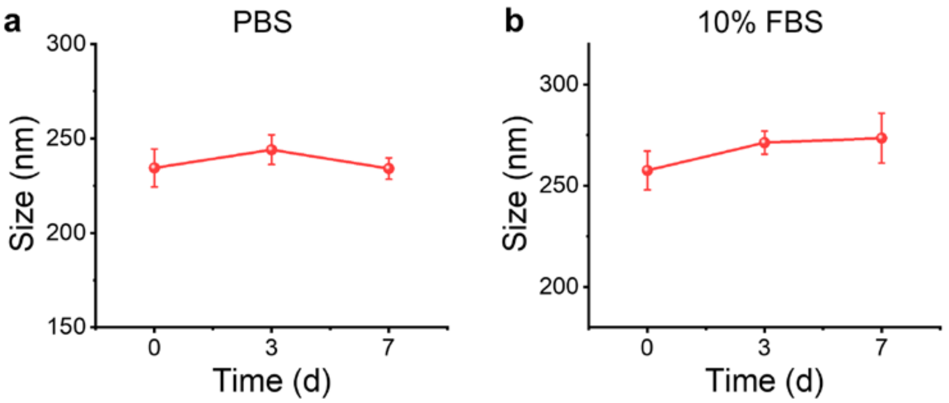

Supplementary Figure 30. Hydrodynamic diameter of **Ru1085** NPs incubated with PBS (a) and 10% FBS (b) for various time. Data are presented as mean ± s.d. (n = 3). Source data are provided as a Source Data file.

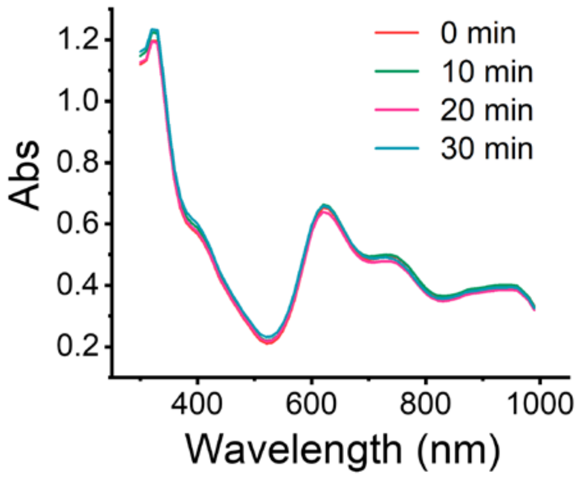

Supplementary Figure 31. Absorption spectra of **Ru1085** NPs with 808 nm laser illumination (0.4 W cm^{-2}) for various time.

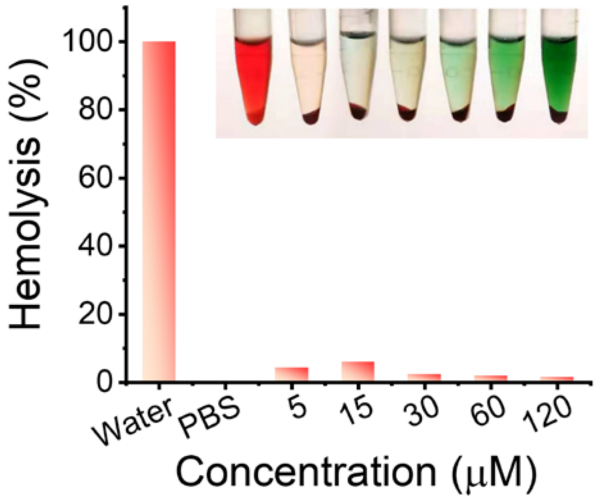

Supplementary Figure 32. Hemolysis tests of **Ru1085** NPs at various concentration (5, 15, 30, 60, and 120 μM).

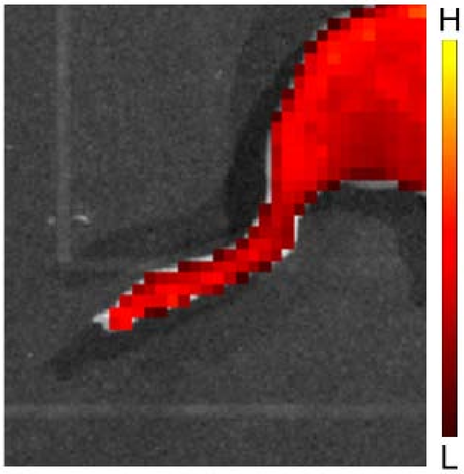

Supplementary Figure 33. Fluorescence images of hindlimb vessels after intravenous injection of $Ru(bpy)_3Cl_2$ NPs (λ_{ex} = 440 nm, and λ_{em} = 620 ± 20 nm).

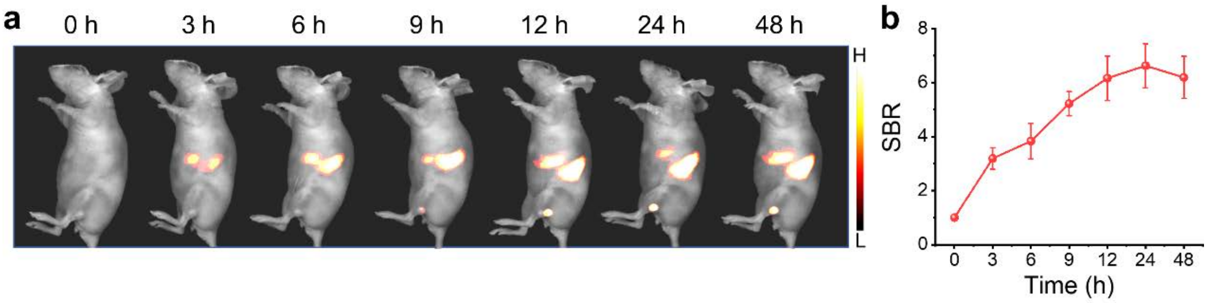

Supplementary Figure 34. NIR-II fluorescence images (a) and signal to background ratio (SBR) (b) of xenografted A549 tumor mice models after intravenous injection (0, 3, 6, 9, 12, 24, 48 h) of **Ru1085** NPs. Data are presented as mean ± s.d. (n = 3 independent mice). Source data are provided as a Source Data file.

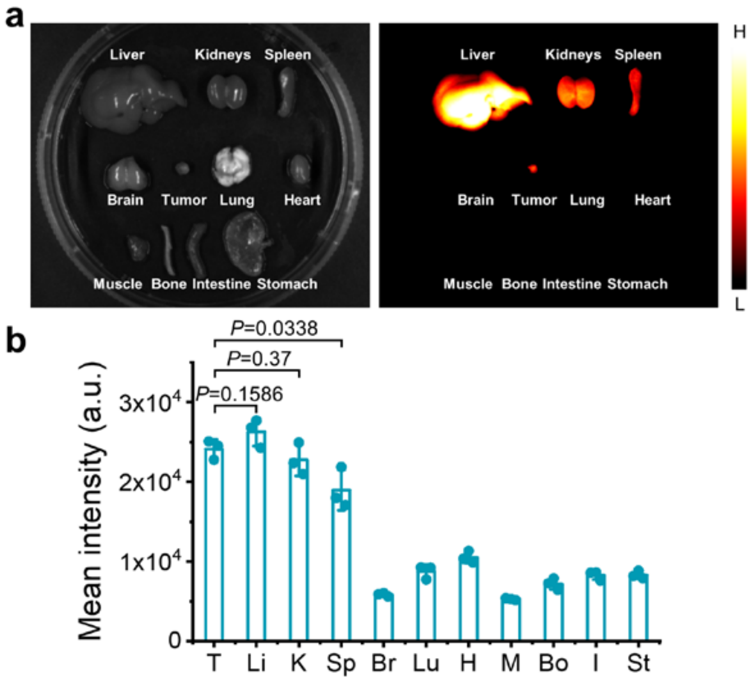

Supplementary Figure 35. Ex vivo NIR-II fluorescence images (a) and analysis the NIR-II FL intensity (b) of tumor (T), liver (Li), kidneys (K), spleen (Sp), brain (Br), lung (Lu), heart (H), muscle (M), bone (Bo), intestine (I) and stomach (St) harvested from xenografted tumor mice models at 24 h post-injection of **Ru1085** NPs. Data are presented as mean ± s.d. (n = 3). Statistical differences were analyzed by Student's two-sided t-test. Source data are provided as a Source Data file.

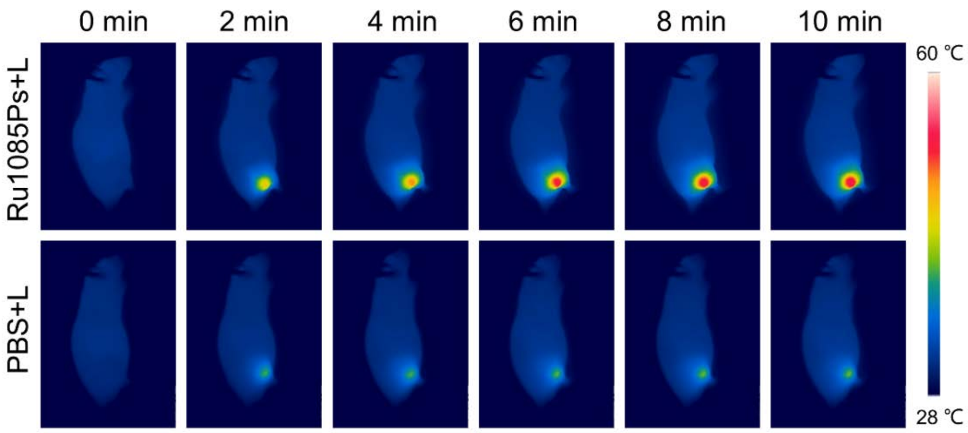

Supplementary Figure 36. IR thermal images of xenografted tumor mice models with 808 nm laser illumination (0.8 W cm^{-2}, 10 min) at 24 h post-injection of **Ru1085** NPs.

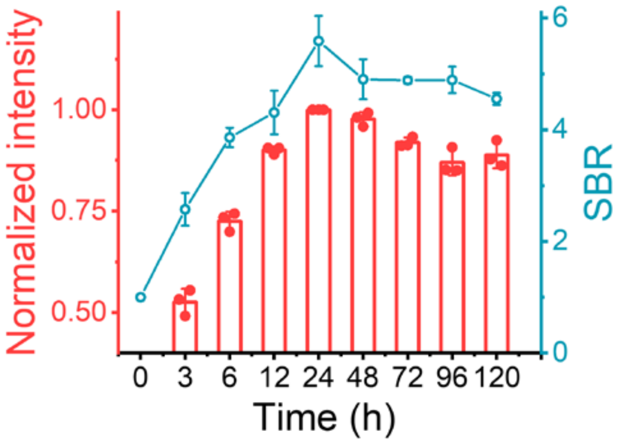

Supplementary Figure 37. Analysis of NIR-II FL intensity (left Y-axis) and SBR (right Y-axis) from tumor based on the images in Fig. 6g. Data are presented as mean ± s.d. (n = 3 independent mice). Source data are provided as a Source Data file.

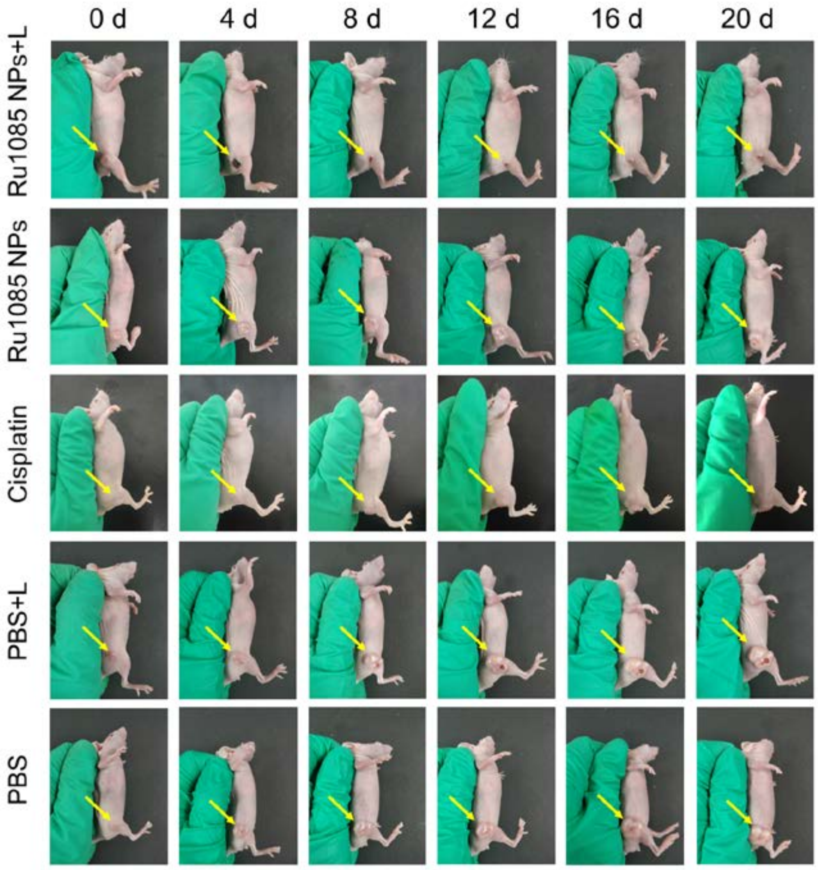

Supplementary Figure 38. Representative photographs of mice with various treatments at different days. Yellow arrow points to the tumor sites (**Ru1085** NPs dose: 1 mg Ru/kg; cisplatin dose: 1 mg Pt/kg; laser treatments: 0.8 W cm^{-2}, 10 min).

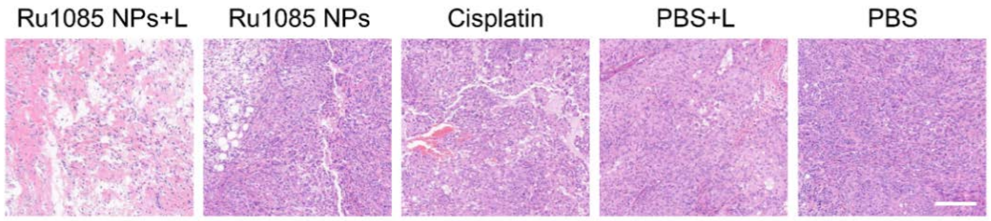

Supplementary Figure 39. H&E staining of tumor tissue slices collected from tumor mice models at 20 days post-injection of **Ru1085** NPs (1 mg Ru/kg), cisplatin (1 mg Pt/kg) or PBS. Scale bar, 200 µm.

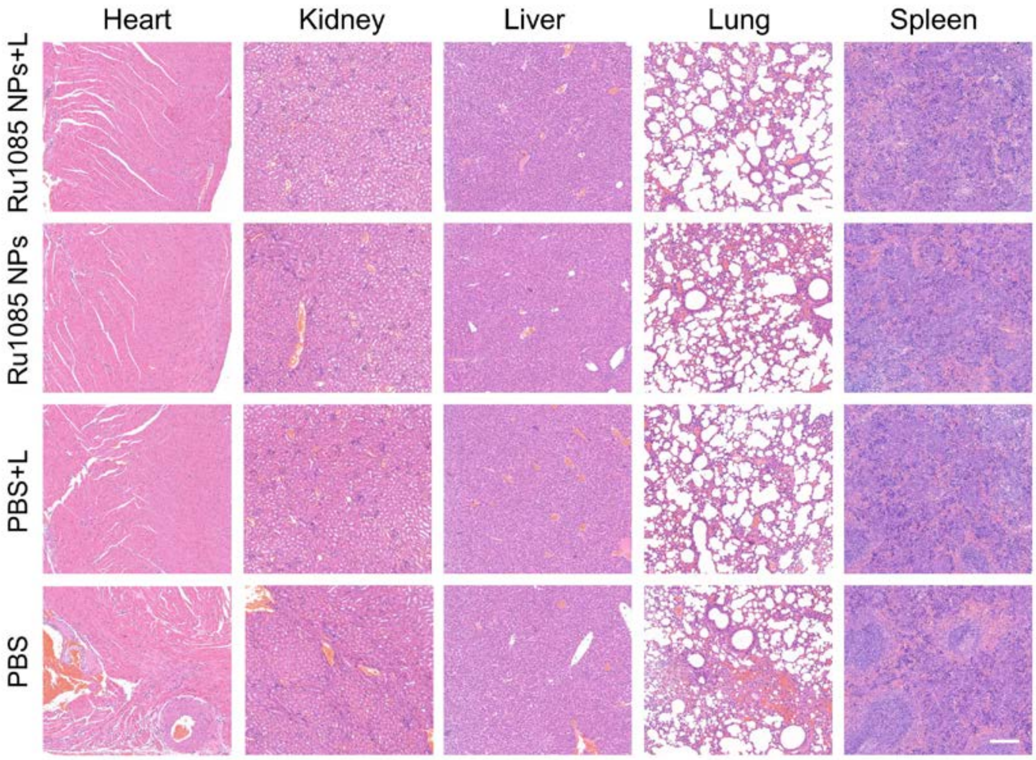

Supplementary Figure 40. H&E staining of the heart, kidney, liver, lung and spleen tissue slices collected from tumor mice models at 20 days post-injection of **Ru1085** NPs (1 mg Ru/kg) or PBS. Scale bar, 200 µm.

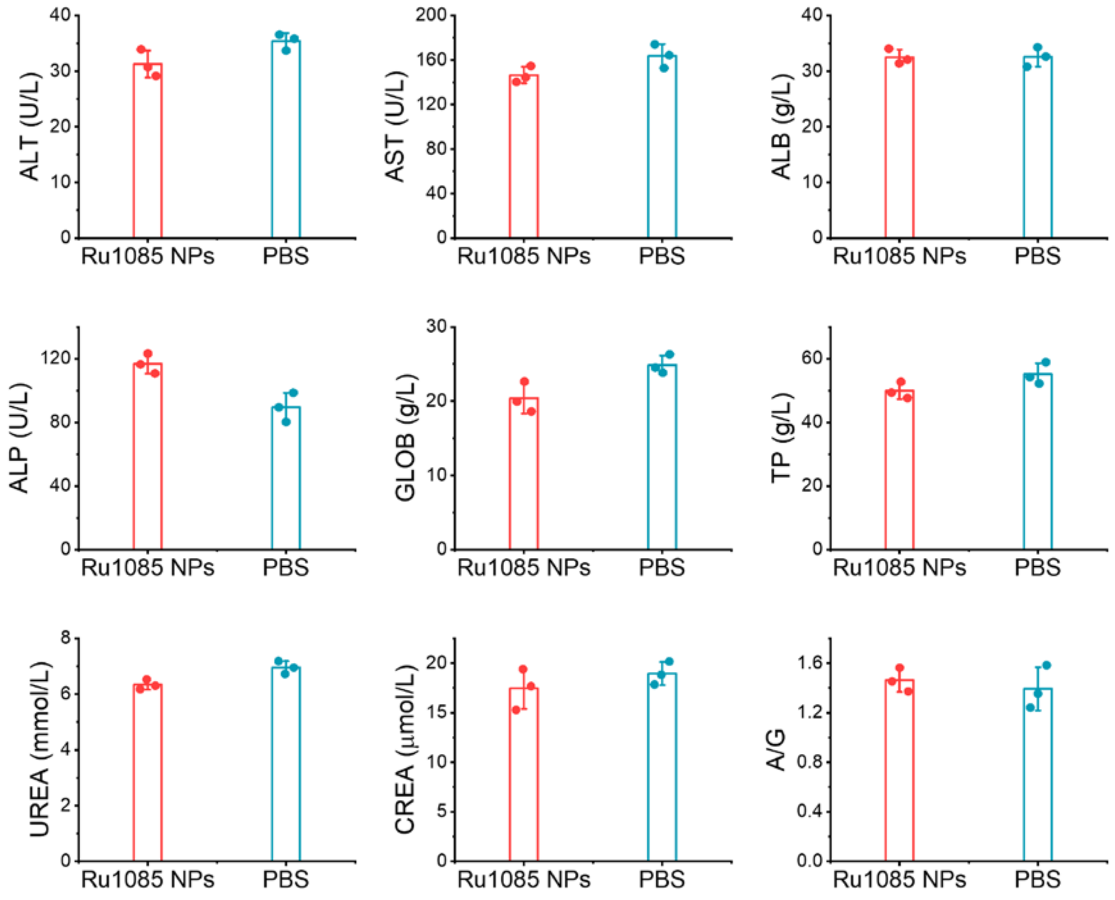

Supplementary Figure 41. Blood chemistry tests at 20 days post-injection of **Ru11085** NPs (1 mg Ru/kg) or PBS (*n* = 3). Data are presented as mean ± s.d. (*n* = 3 independent samples). Source data are provided as a Source Data file.

4. Synthetic Procedures and Characterization Data

a) Synthesis of compound 1

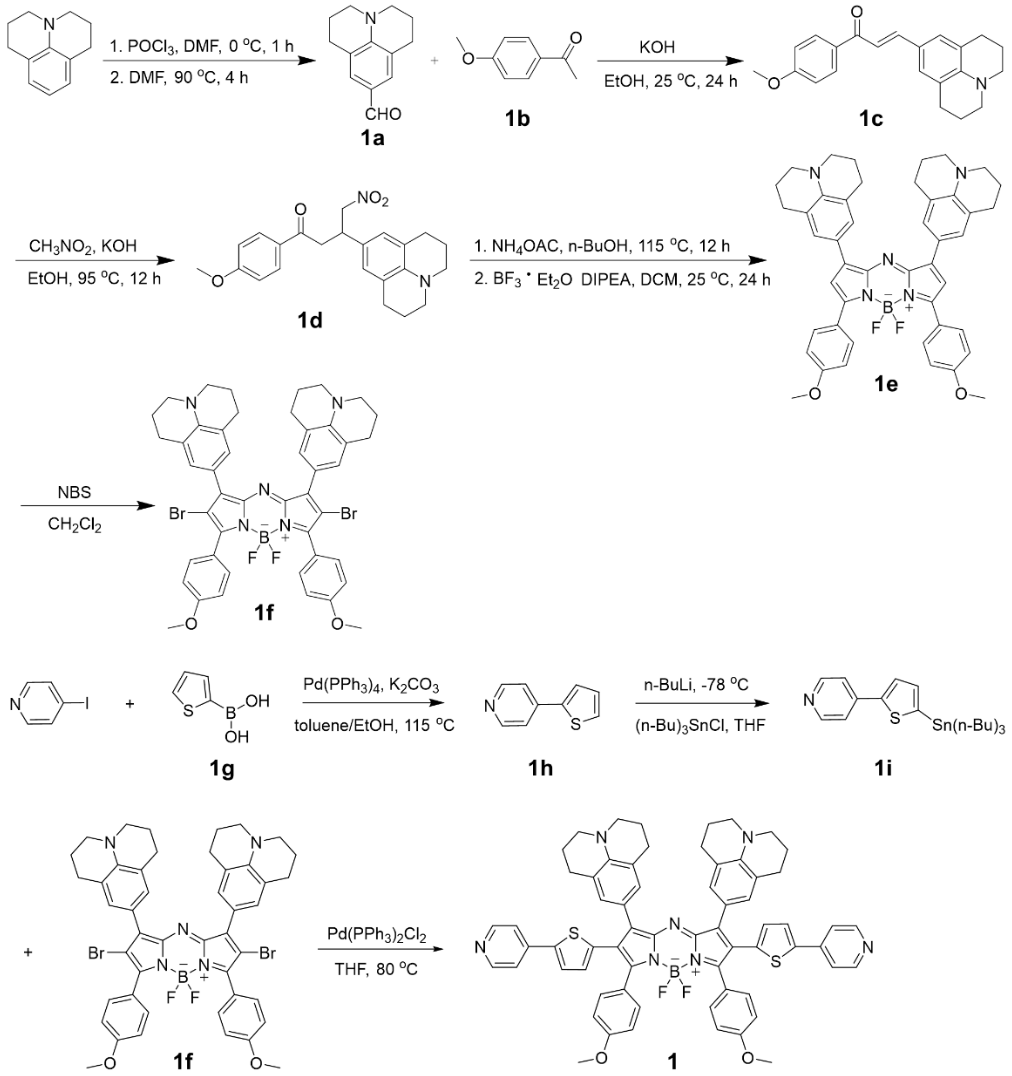

Supplementary Figure 42. The Synthesis of **1.**

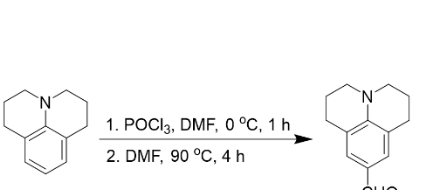

Synthesis of compound ***1a***[1]*:*

Freshly distilled phosphorus oxychloride ($POCl_3$) (0.75 mL) and N, N-Dimethylformamide (DMF) (0.75 mL) were mixed at 0 °C under the protection of N_2 atmosphere and the reaction mixture was stirred for 1 h at room temperature. Julolidine (2.0 g, 11.54 mmol) was added dropwise in DMF (10

mL), followed by stirred at 90 °C for 4 h. After treated with saturated sodium bicarbonate ($NaHCO_3$) solution at room temperature for 2 h, the reaction mixture was extracted with ethyl acetate (EA) and dried with anhydrous sodium sulfate (Na_2SO_4). The crude product was purified by silica gel chromatography using petroleum ether (PE) and EA (PE : EA = 10 : 1) to obtain yellow solid **1a** (1.97 g, 85%). ^{1}H NMR (400 MHz, $CDCl_3$) δ: 9.58 (s, 1H), 7.28 (s, 2H), 3.30-3.26 (m, 4H), 2.76 (t, J = 6.2 Hz, 4H), 1.94 (dd, J = 11.8, 6.0 Hz, 4H). ^{13}C NMR (100 MHz, $CDCl_3$) δ: 190.13, 147.87, 129.48, 123.94, 120.29, 50.02, 27.64, 21.23.

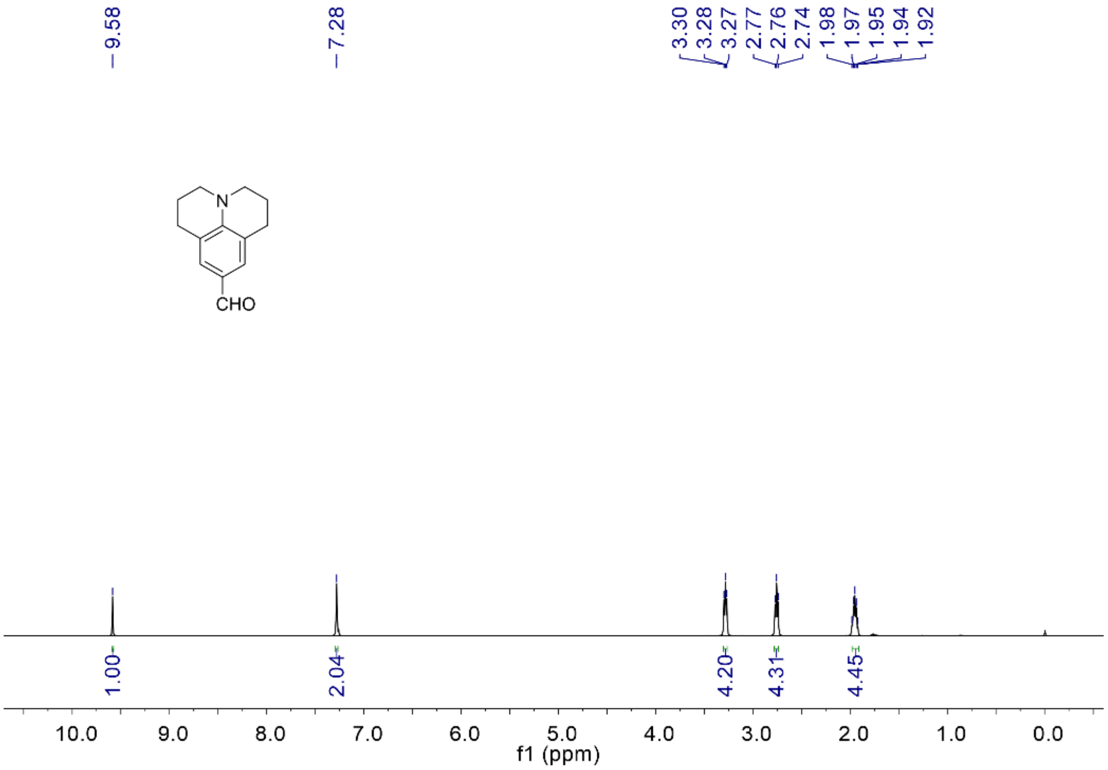

Supplementary Figure 43. ^{1}H NMR spectrum of **1a**.

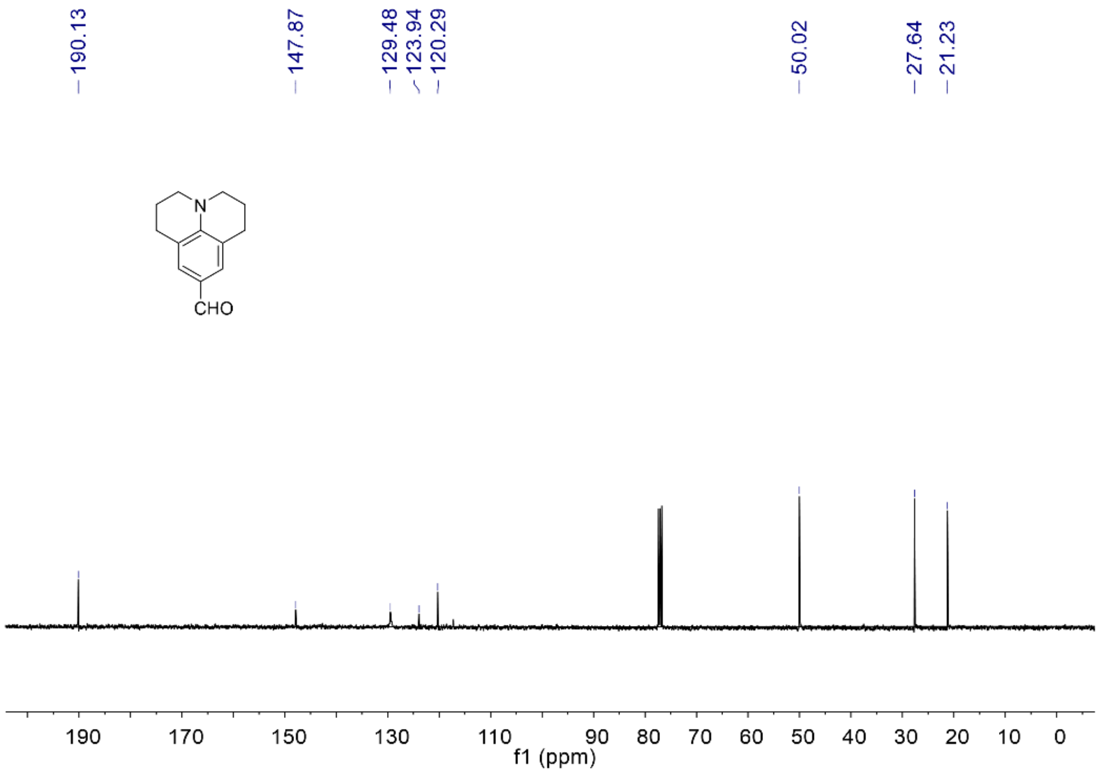

Supplementary Figure 44. ^{13}C NMR spectrum of **1a**.

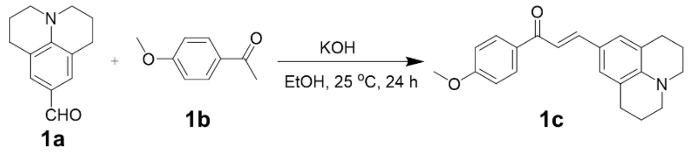

*Synthesis of compound **1c**[1]:*

1a (2 g, 9.94 mmol) and **1b** (1.49 g, 9.94 mmol) were dissolved in ethanol (EtOH) (60 mL). After adding potassium hydroxide (KOH) (2.75 g, 49.68 mmol) and H_2O (15 mL), the reaction mixture was stirred at room temperature for 24 h. Then, excess EtOH was removed under reduced pressure. The remaining aqueous solution was diluted with brine, extracted with EA, and dried with anhydrous Na_2SO_4. Then, the crude residue was concentrated via rotary evaporation, followed by purification with silica gel column chromatograph using PE and EA (PE: EA = 15:1) to obtain red solid **1c** (2.13 g, 65%). ^{1}H NMR (400 MHz, $CDCl_3$) δ: 8.02 (d, J = 8.8 Hz, 2H), 7.70 (d, J = 15.3 Hz, 1H), 7.26 (s, 1H), 7.11 (s, 2H), 6.96 (d, J = 8.8 Hz, 2H), 3.87 (s, 3H), 3.28 – 3.18 (m, 4H), 2.76 (t, J = 6.3 Hz, 4H), 1.99 – 1.92 (m, 4H). ^{13}C NMR (100 MHz, $CDCl_3$) δ: 188.66, 162.64, 145.34, 144.95, 131.90, 130.30, 127.89, 121.56, 120.81, 115.25, 113.43, 55.28, 49.77, 27.53, 21.41.

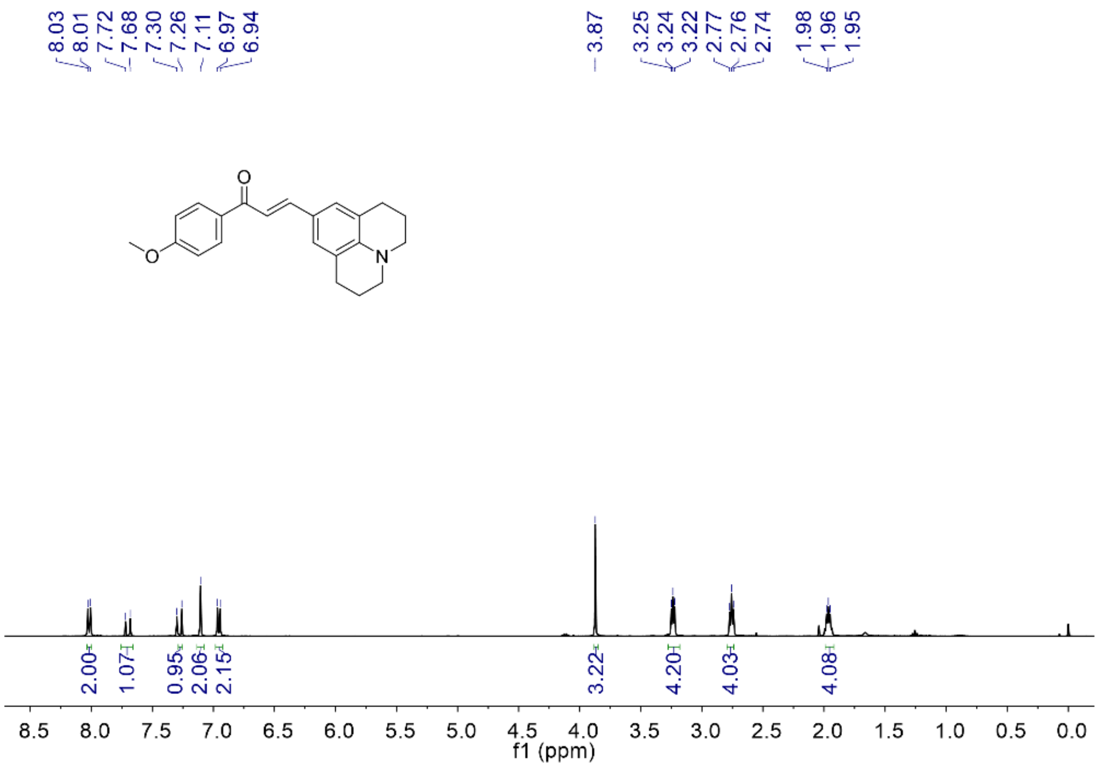

Supplementary Figure 45. ^{1}H NMR spectrum of **1c**.

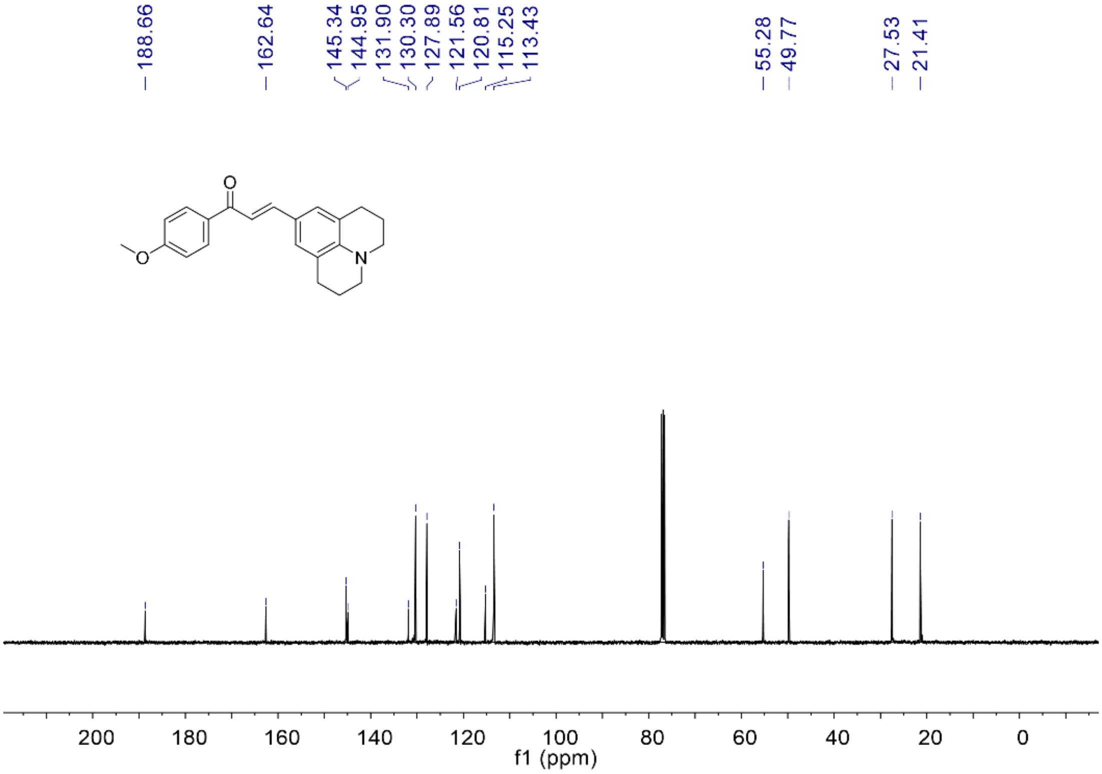

Supplementary Figure 46. ^{13}C NMR spectrum of **1c**.

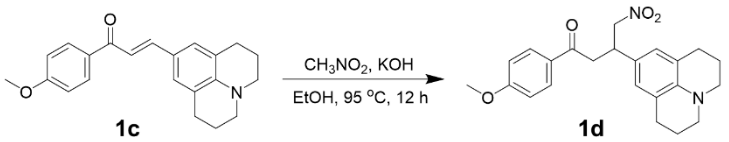

Synthesis of compound ***1d***[1]*:*

Nitromethane (CH_3NO_2) (2.75 g, 45.05 mmol) and KOH (0.25 g, 4.50 mmol) was added to a vigorously stirred solution of **1c** (1.0 g, 3.00 mmol) in EtOH (10 mL). After stirring and refluxing at 95 °C for 12 h. Then, excess EtOH was removed under reduced pressure. The remaining aqueous solution was diluted with brine, extracted with EA, and dried with anhydrous Na_2SO_4. Then, the crude residue was concentrated via rotary evaporation, followed by purification via silica gel column chromatograph using PE and EA (PE : EA = 10 : 1) to obtain **1d** (0.82 g, 70%). ^{1}H NMR (400 MHz, $CDCl_3$) δ: 7.89 (d, J = 8.7 Hz, 2H), 6.89 (d, J = 8.7 Hz, 2H), 6.64 (s, 2H), 4.72 (dd, J = 12.2, 6.7 Hz, 1H), 4.56 (dd, J = 12.2, 8.1 Hz, 1H), 3.97 (dt, J = 14.2, 7.1 Hz, 1H), 3.83 (s, 3H), 3.29 (dt, J = 17.4, 10.5 Hz, 2H), 3.10 – 3.05 (m, 4H), 2.68 (t, J = 6.4 Hz, 4H), 1.91 (dt, J = 11.9, 6.1 Hz, 4H). ^{13}C NMR (100 MHz, $CDCl_3$) δ: 195.92, 163.55, 142.25, 130.25, 129.51, 125.83, 125.67, 121.68, 113.66, 79.90, 55.36, 49.75, 41.45, 38.60, 27.49, 21.79.

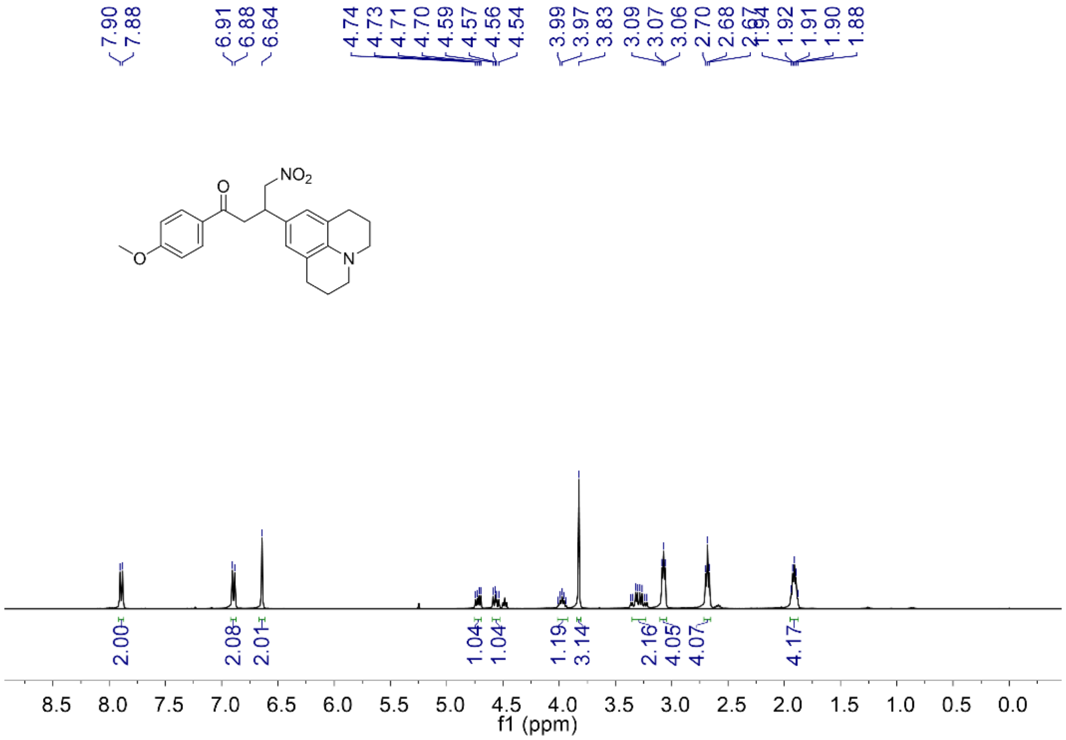

Supplementary Figure 47. ^{1}H NMR spectrum of **1d**.

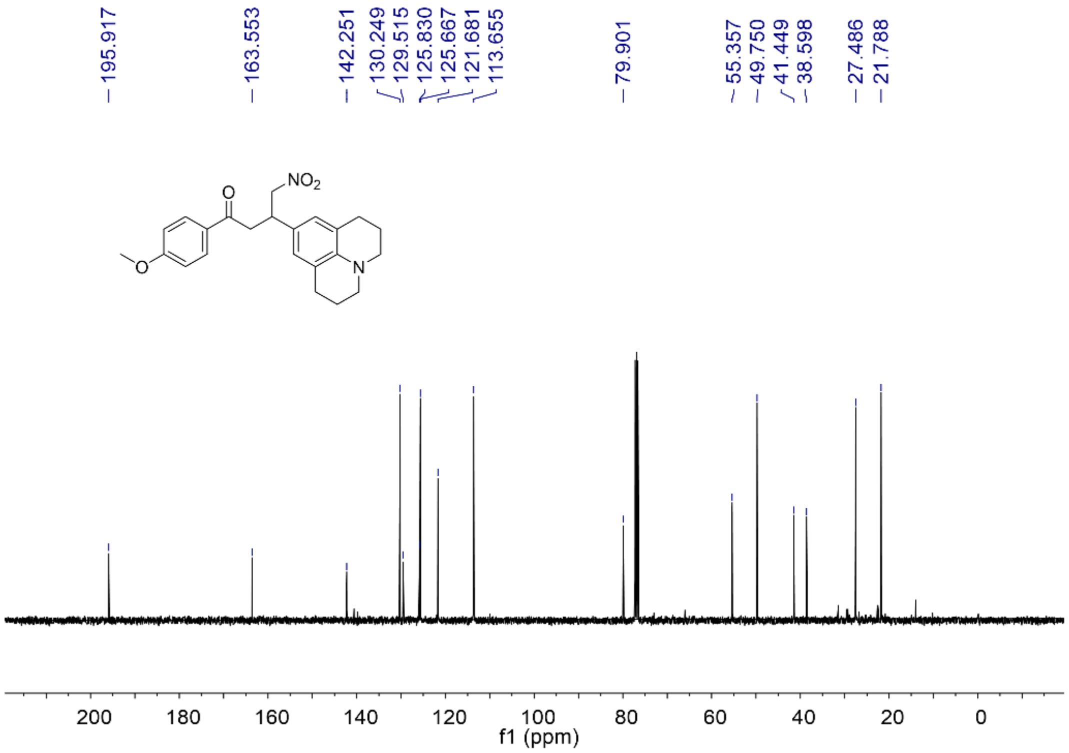

Supplementary Figure 48. ^{13}C NMR spectrum of **1d**.

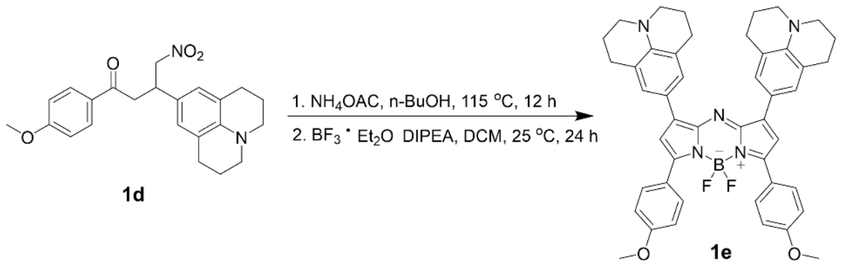

Synthesis of compound ***1e***[1]*:*

1d (0.85 g, 2.15 mmol) and ammonium acetate (2.49 g, 32.25 mmol) was dissolved in *n*-butanol (20 mL). After stirring at 115 °C for 12 h, the mixture was cooled to room temperature, concentrated by rotary evaporation and washed three times with ethanol to remove impurities to obtain dark blue film. Then the dark blue film (1 equiv.), anhydrous N, N-Diisopropylethylamine (DIPEA) (10 equiv.) and boron trifluoride etherate (15 equiv.) were dissolved in DCM (15 mL). After stirring at room temperature for 24 h under the protection of N_2 atmosphere, the mixture was diluted with water, extracted with DCM, dried with anhydrous Na_2SO_4. Further, the crude product was concentrated via rotary evaporation and purified with silica gel column chromatography using PE and DCM (PE : DCM

= 1 : 1) as the eluent to obtain brown solid **1e** (0.56 g, 70%). ^{1}H NMR (400 MHz, $CDCl_3$) δ: 7.89 (d, J = 8.5 Hz, 4H), 7.60 (s, 4H), 7.04 (d, J = 8.5 Hz, 4H), 6.94 (s, 2H), 3.91 (s, 6H), 3.26 – 3.18 (m, 8H), 2.80 (t, J = 6.2 Hz, 8H), 2.02 (dt, J = 11.5, 5.9 Hz, 8H). ^{13}C NMR (100 MHz, $CDCl_3$) δ: 160.70, 153.29, 149.54, 142.81, 142.75, 127.92, 127.85, 125.75, 122.20, 121.08, 114.43, 110.84, 55.48, 50.16, 27.91, 22.18.

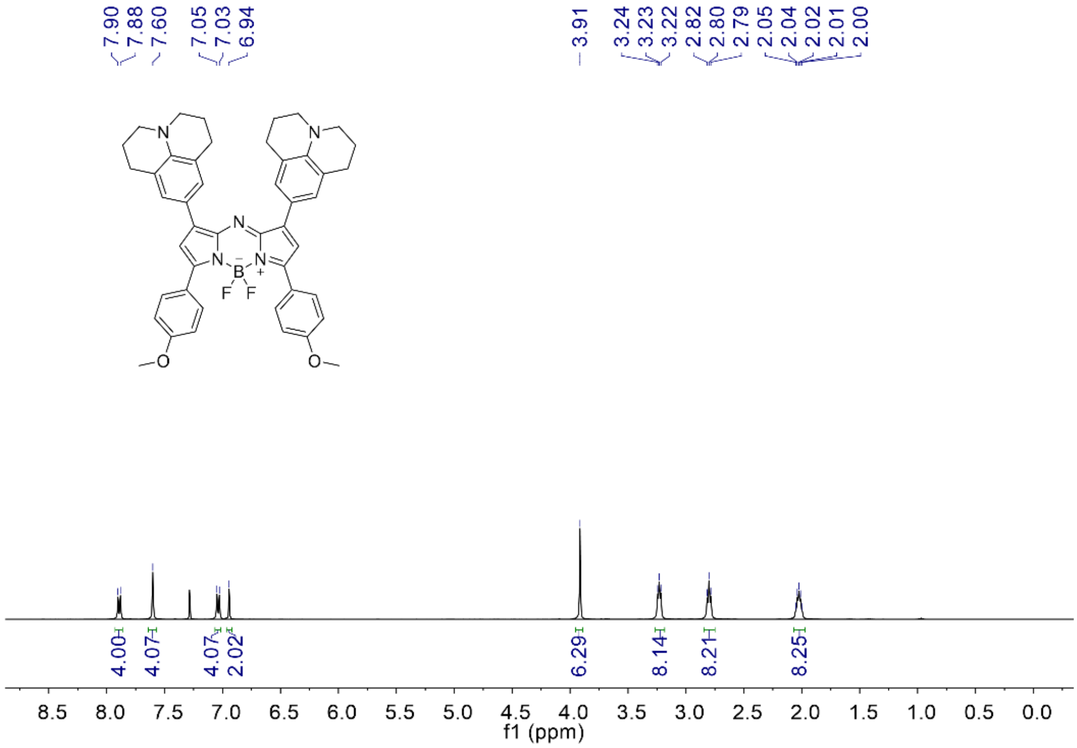

Supplementary Figure 49. ^{1}H NMR spectrum of **1e**.

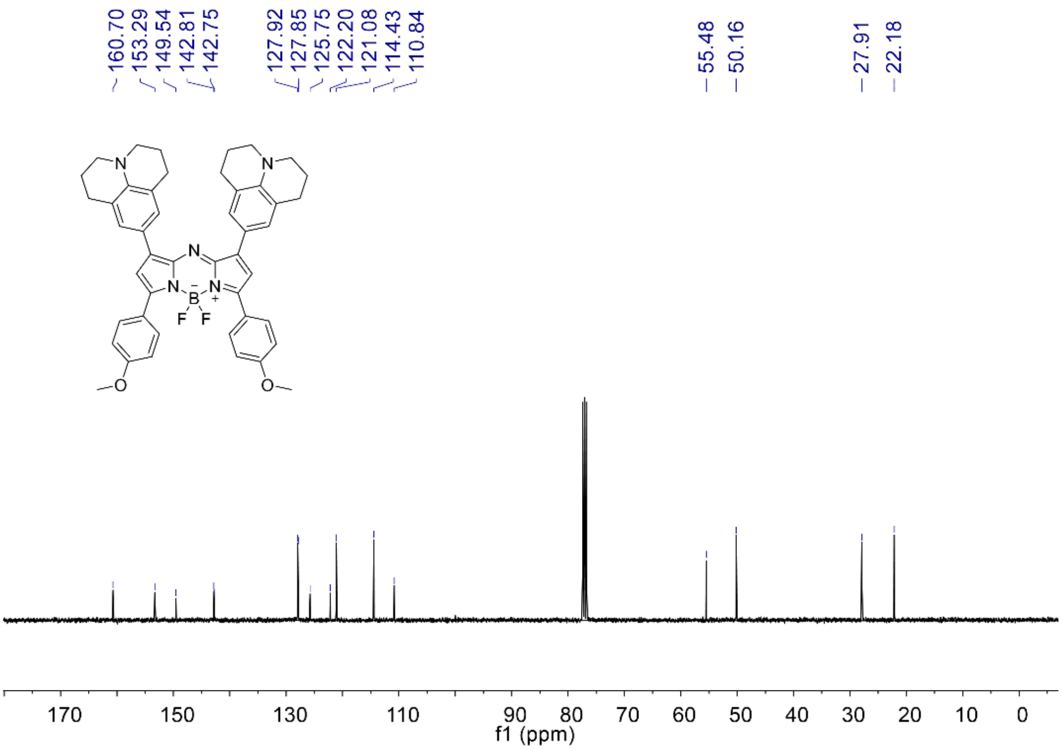

Supplementary Figure 50. ^{13}C NMR spectrum of **1e**.

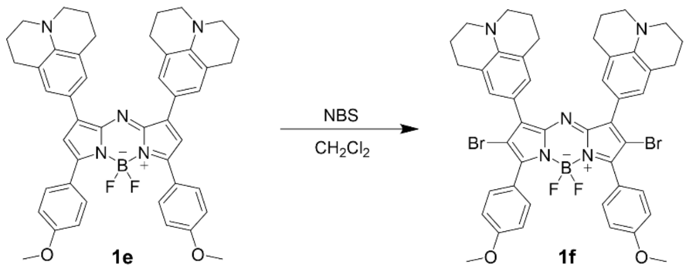

*Synthesis of compound **1f**:*

1e (56 mg, 0.074 mmol) was dissolved in DCM, and then the mixture was added N-Bromosuccinimide (NBS) (28.9 mg, 0.16 mmol) under the condition of an ice bath. The reaction was allowed to carry out for 15 min. After reaction, the mixture was stood to room temperature, diluted with DCM, washed with saturated ammonium chloride (NH_4Cl) solution, water and saturated aqueous brine, followed by dried with anhydrous Na_2SO_4. Further, the crude product was concentrated via rotary evaporation and purified with silica gel column chromatography using PE and DCM (PE : DCM = 1 : 1) as the eluent to obtain yellow oil **1f** (54.9 mg, 82%). ^1H NMR (400 MHz, $CDCl_3$) δ: 7.68 (d, J = 8.7 Hz, 4H), 7.52 (s, 4H), 6.94 (d, J = 8.7 Hz, 4H), 3.83 (s, 6H), 3.27 - 3.23 (m, 8H), 2.78 (t, J = 6.2 Hz, 8H), 2.01 - 1.96 (m, 8H). ^{13}C NMR (100 MHz, $CDCl_3$) δ: 160.85, 144.21, 132.13, 130.14, 122.88, 120.61, 119.05, 113.26, 55.18,

50.07, 27.74, 21.80. MALDI-TOF/MS (m/z): Calcd. for $[C_{46}H_{42}BBr_2F_2N_5O_2]^+$: 903.1766. Found: 903.2162.

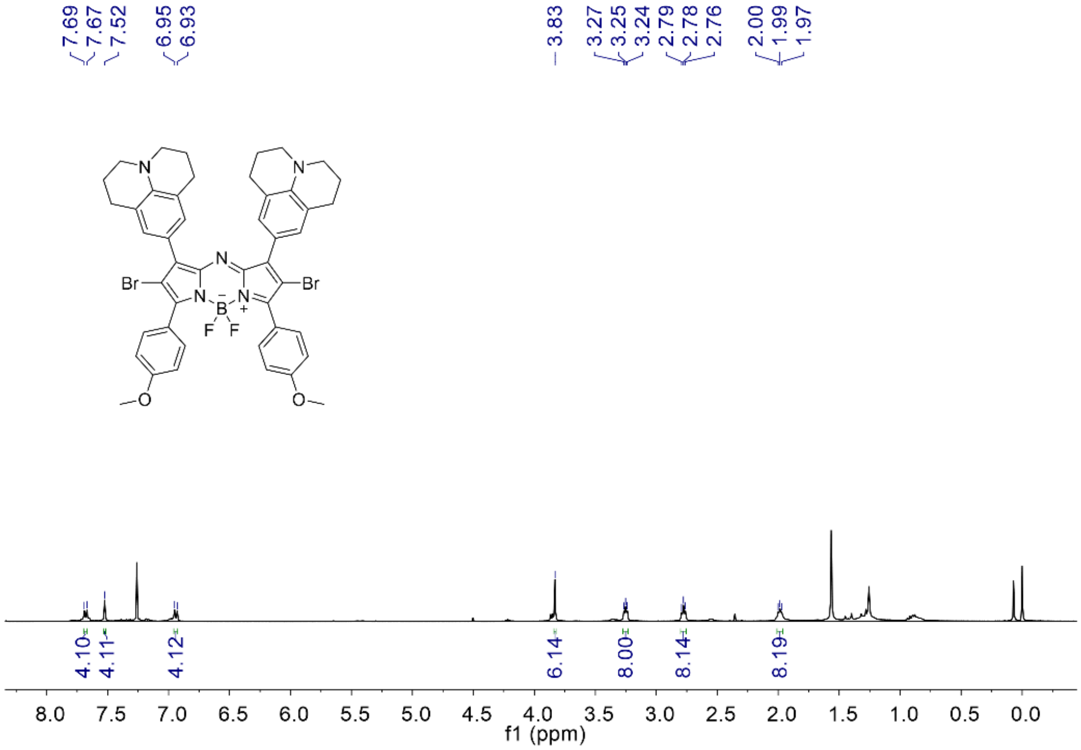

Supplementary Figure 51. ^1H NMR spectrum of **1f**.

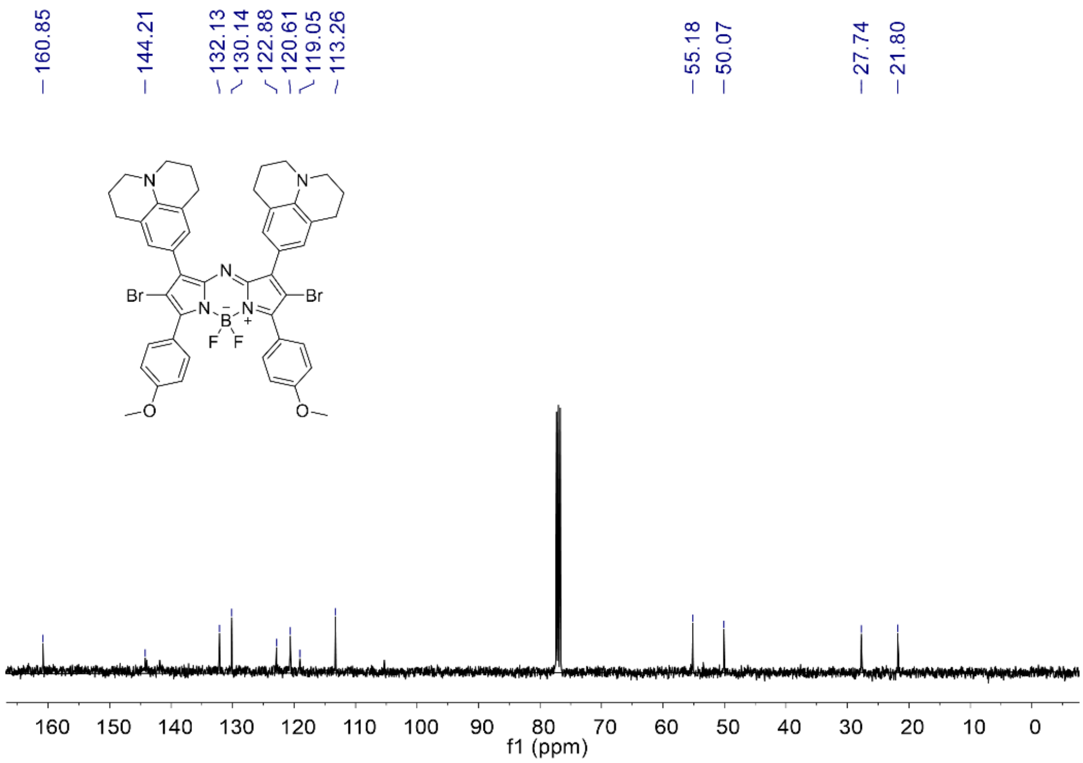

Supplementary Figure 52. ^{13}C NMR spectrum of **1f**.

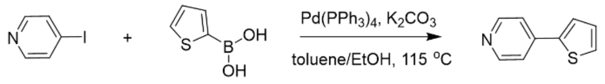

1g **1h**

.

Synthesis of compound **1h**[2]:

4-Iodopyridine (2.05 g, 10.00 mmol), **1g** (1.41 g, 11.01 mmol) and potassium carbonate (K_2CO_3) (1.72 g, 12.45 mmol) were dissolved in a mixture of toluene and EtOH (toluene : EtOH = 3 : 1), followed by added tetrakis(triphenylphosphine)palladium ($Pd(PPh_3)_4$) (1.16 g, 1.00 mmol). After refluxing at 115 °C for overnight, the reaction was concentrated via rotary evaporation and purified with silica gel column chromatography using hexane and DCM (hexane : DCM = 9 : 1) as the eluent to obtain white solid **1h** (1.21 g, 75%). ^{1}H NMR (400 MHz, $CDCl_3$) δ: 8.63 (d, J = 4.8 Hz, 2H), 7.56 - 7.54 (m, 1H), 7.53 - 7.50 (m, 2H), 7.46 (d, J = 5.0 Hz, 1H), 7.18 (t, J = 4.3 Hz, 1H). ^{13}C NMR (100 MHz, $CDCl_3$) δ: 150.34, 141.41, 141.14, 128.46, 127.25, 125.38, 119.91.

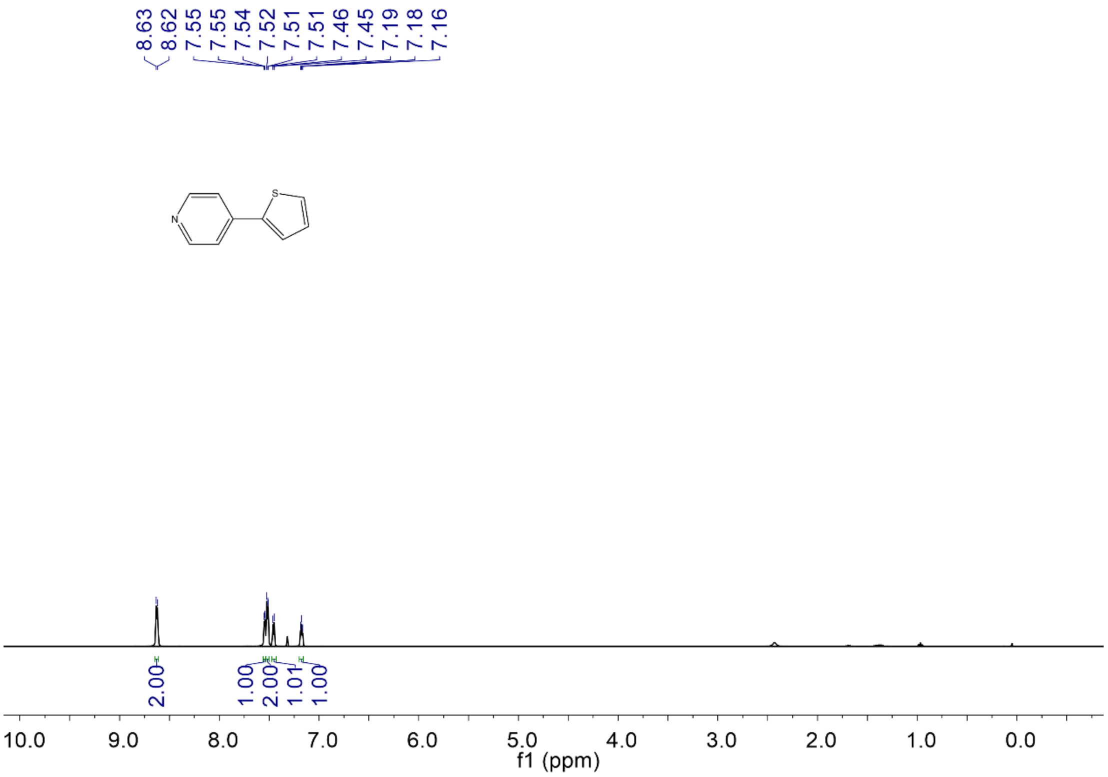

Supplementary Figure 53. ^{1}H NMR spectrum of **1h**.

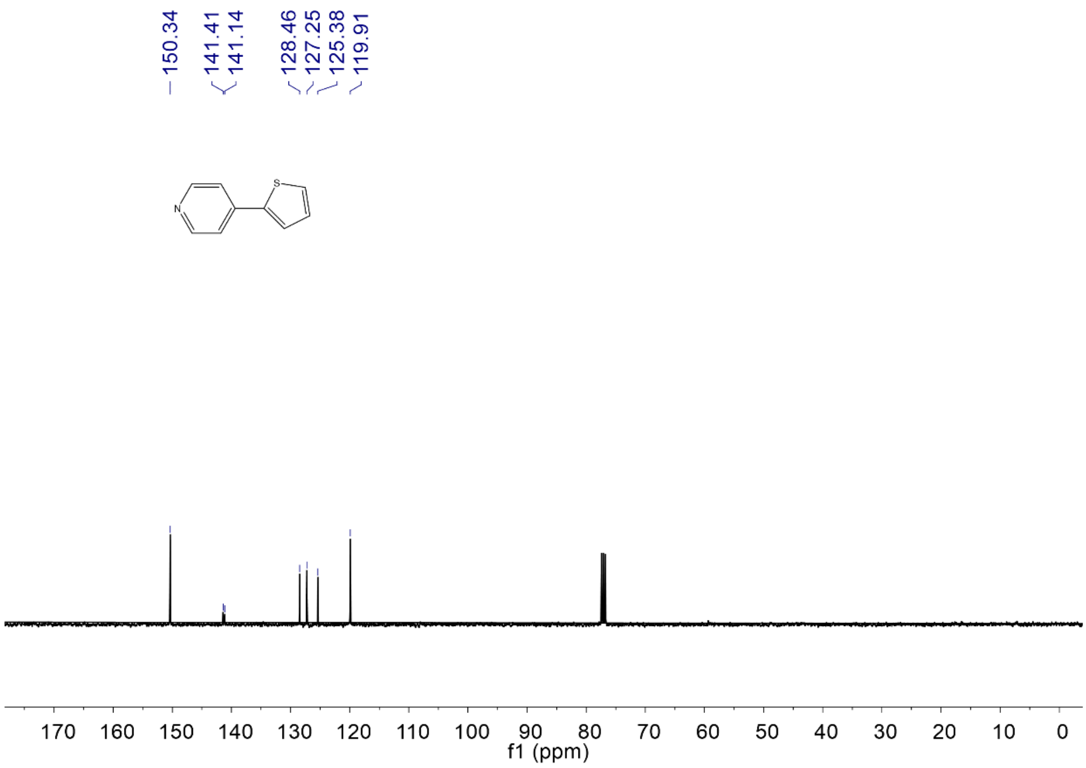

Supplementary Figure 54. ^{13}C NMR spectrum of **1h.**

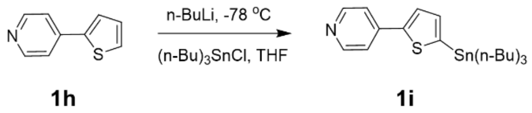

*Synthesis of compound **1i**[2]:*

n-BuLi (0.22 g, 3.43 mmol) was added dropwise to a vigorously stirred solution of **1h** (0.18 g, 1.13 mmol) in THF at -78 °C. The reaction mixture was stored at this temperature for 30 min before adding $(n\text{-}Bu)_3SnCl$ (404.6 mg, 1.24 mmol) dropwise. The resulting mixture was allowed to stir at room temperature for 12 h. The reaction was extracted with saturated NH_4Cl and EA. The organic layers were washed with water, followed by dried with anhydrous Na_2SO_4. Further, the crude product was concentrated via rotary evaporation to obtain red brown oil **1i** (396 mg, 78%). ^1H NMR (400 MHz, $CDCl_3$) δ: 8.56 (d, J = 6.1 Hz, 2H), 7.60 (d, J = 3.4 Hz, 1H), 7.48 (dd, J = 4.7, 1.5 Hz, 2H), 7.19 (d, J = 3.4 Hz, 1H), 1.59 (dt, J = 15.6, 7.7 Hz, 6H), 1.36 (dd, J = 14.8, 7.4 Hz, 6H), 1.19 - 1.09 (m, 6H), 0.91 (t, J = 7.3 Hz, 9H). ^{13}C NMR (100 MHz, $CDCl_3$) δ: 150.25, 146.57, 141.43, 140.56, 136.70, 126.36, 119.88, 28.93, 27.25, 13.66, 10.93.

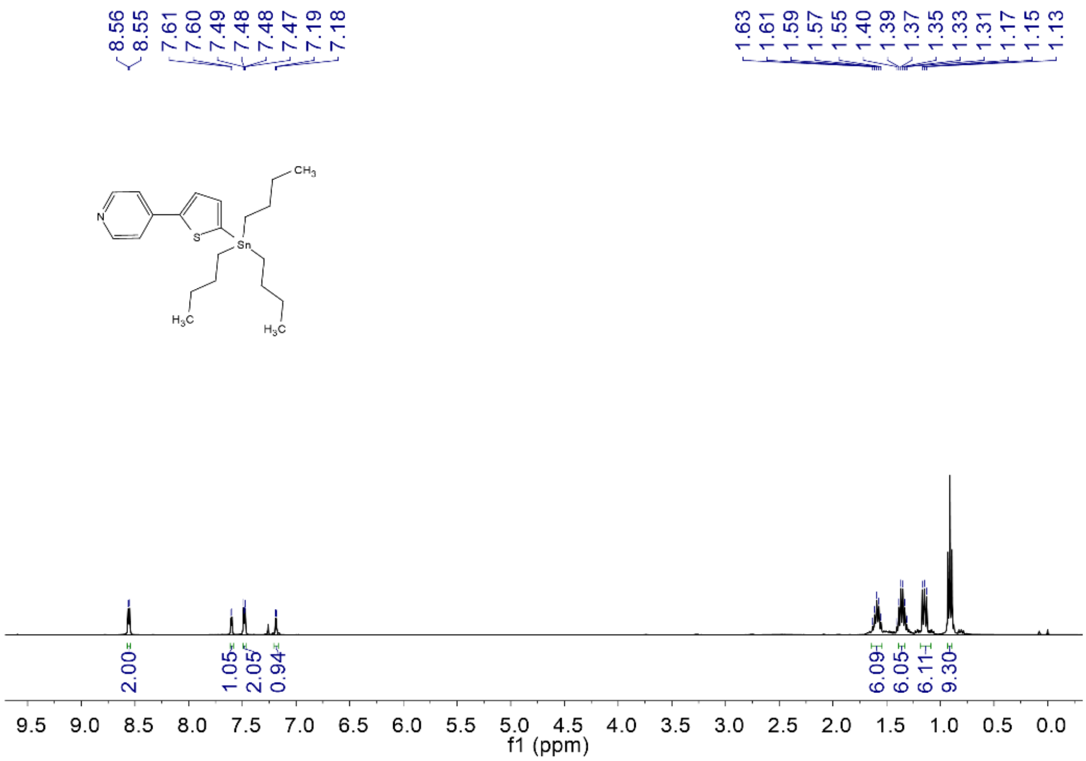

Supplementary Figure 55. ^1H NMR spectrum of **1i**.

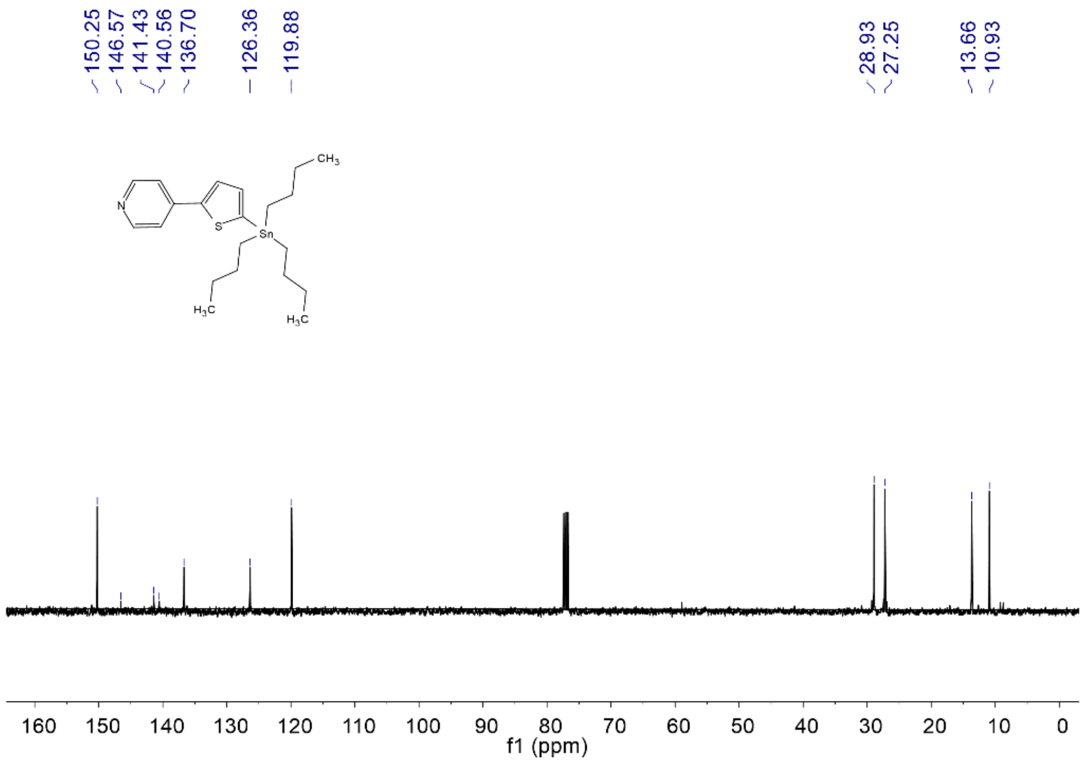

Supplementary Figure 56. ^{13}C NMR spectrum of **1i.**

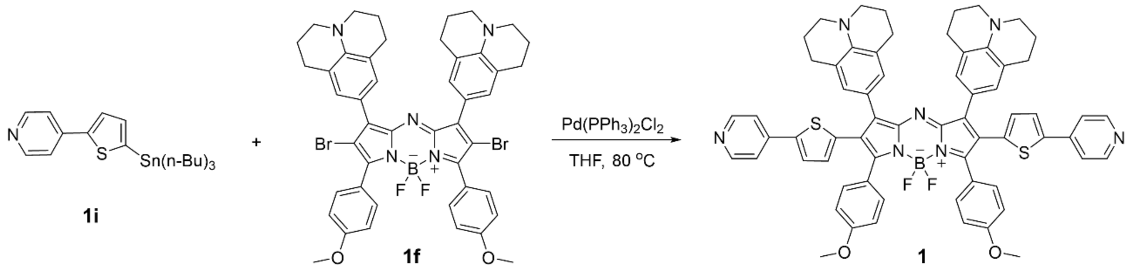

*Synthesis of compound **1**:*

1i (21.87 mg, 0.049 mmol), **1f** (20.0 mg, 0.022 mmol) and dichlorobis(triphenylphosphine)palladium(II) ($Pd(PPh_3)_2Cl_2$) (3.4 mg, 0.0048 mmol) were dissolved in THF and the reaction mixture was stirred under N_2 at 80 °C for overnight. After TLC monitored the completed reaction, the reaction mixture was concentrated under rotary evaporation and purified by silica gel column chromatography using EA as the eluent to obtain blue solid **1** (9.84 mg, 42%). ^{1}H NMR (400 MHz, $CDCl_3$) δ: 8.53 (d, J = 6.1 Hz, 4H), 7.47 (d, J = 8.8 Hz, 4H), 7.37 (d, J = 6.2 Hz, 4H), 7.32 (d, J = 3.7 Hz, 2H), 7.20 (s, 4H), 6.79 (d, J = 8.9 Hz, 4H), 6.70 (d, J = 3.7 Hz, 2H), 3.77 (s, 6H), 3.23 - 3.19 (m, 8H), 2.58 (t, J = 6.3 Hz, 8H), 1.95 - 1.90 (m, 8H). ^{13}C NMR (100 MHz, $CDCl_3$) δ: 160.43, 150.17, 145.09, 143.94, 141.42, 141.14, 138.86, 131.78, 130.47, 130.09, 125.35, 123.29, 120.54, 119.57, 119.50, 113.23, 55.11, 50.06, 27.64, 21.86. ESI-MS (m/z): Calcd. for $[C_{64}H_{54}BF_2N_7O_2S_2+H]^+$: 1066.3842. Found: 1066.3871.

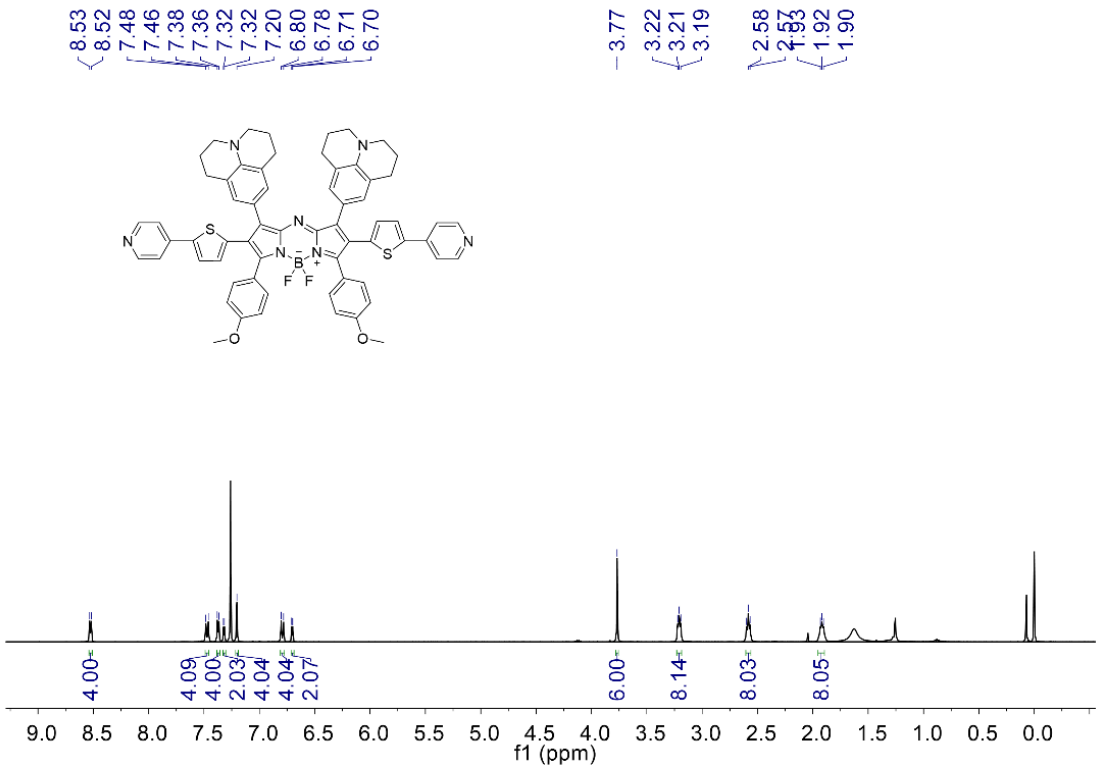

Supplementary Figure 57. ^{1}H NMR spectrum of **1**.

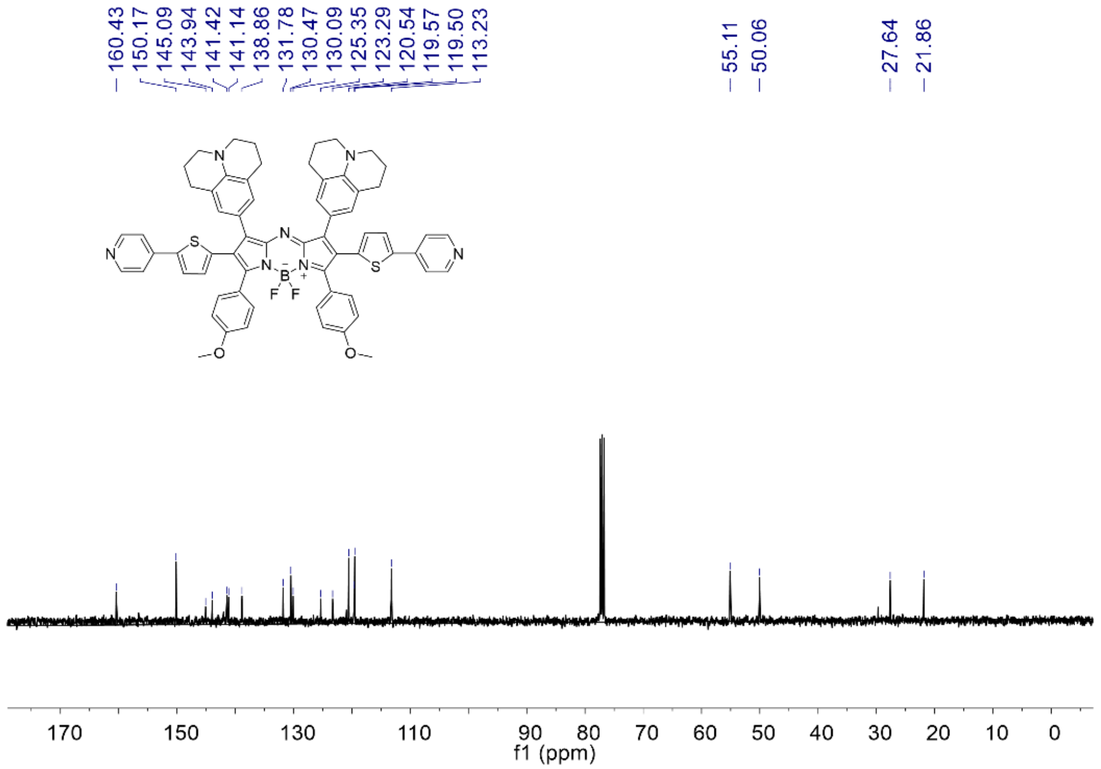

Supplementary Figure 58. ^{13}C NMR spectrum of **1**.

b) Synthesis and Characterization of 2

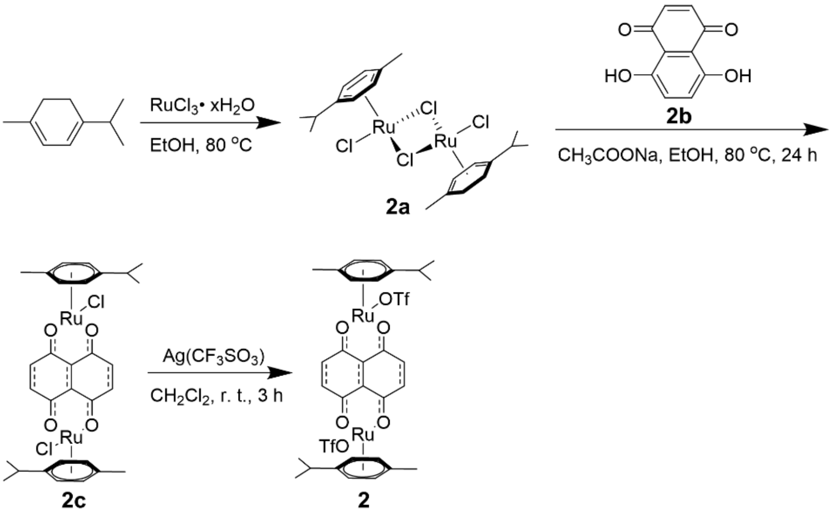

Supplementary Figure 59. The Synthesis of **2.**

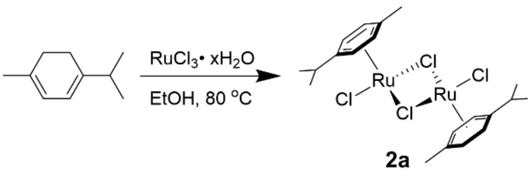

Synthesis of compound ***2a***[3]*:*

Ruthenium (III) chloride trihydrate (2 g, 9.66 mmol) was dissolved in ethanol, and then terpilene (4.57 mL, 3.65 g, 26.8 mmol) was added under the protection of argon atmosphere. After heated at 80 °C for red solid **2a** (2.65 g, 90%). ^{1}H NMR (400 MHz, $CDCl_3$) δ: 5.41 (d, J = 6.0 Hz, 4H), 5.28 (d, J = 6.0 Hz, 4H), 2.09 (s, 6H), 1.63 (s, 2H), 1.21 (d, J = 6.9 Hz, 12H). ^{13}C NMR (100 MHz, $CDCl_3$) δ: 101.10, 96.64, 81.21, 80.45, 30.53, 22.05, 18.83.

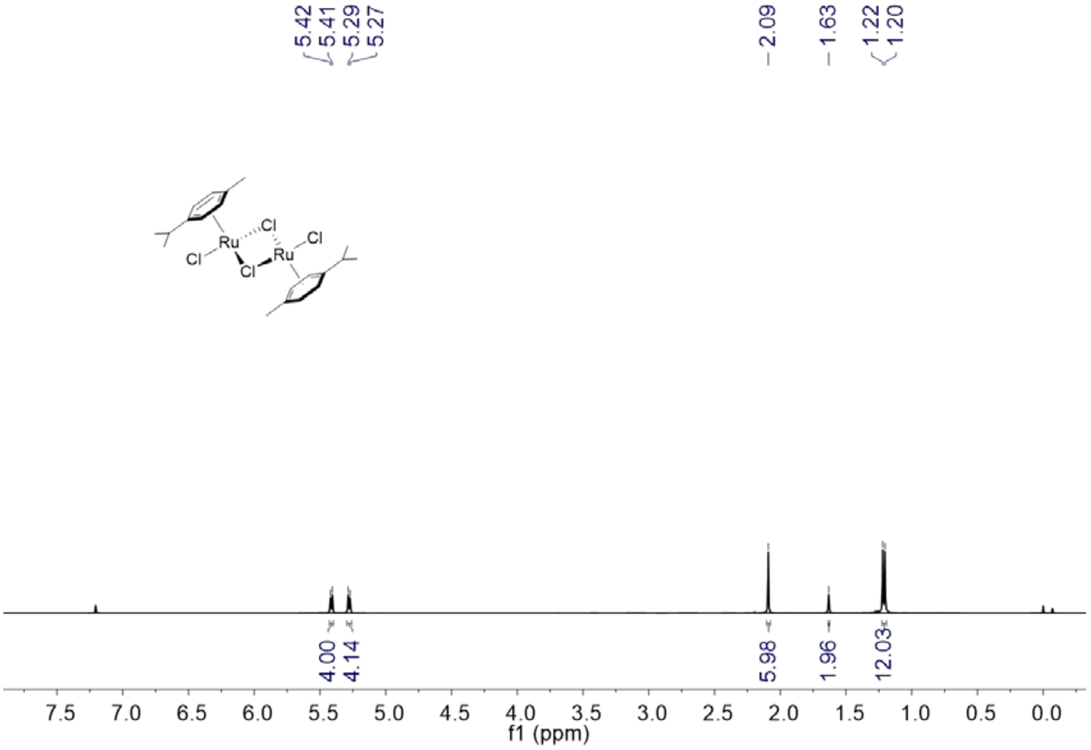

Supplementary Figure 60. ^{1}H NMR spectrum of **2a.**

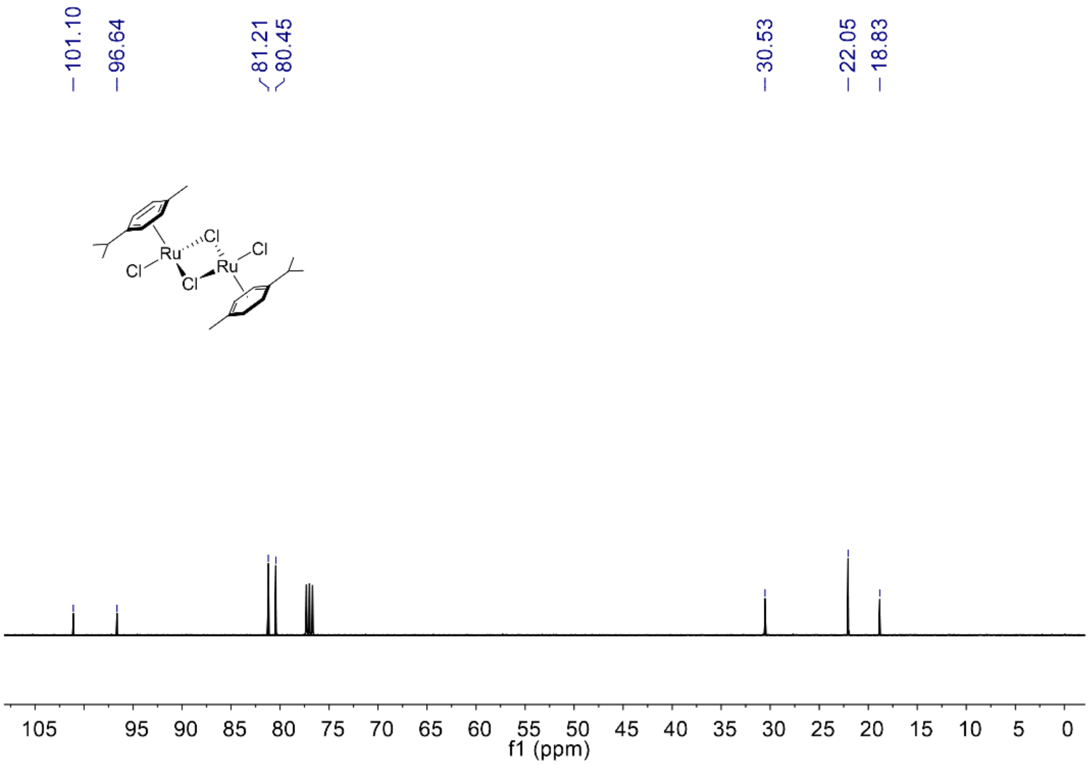

Supplementary Figure 61. ^{13}C NMR spectrum of **2a.**

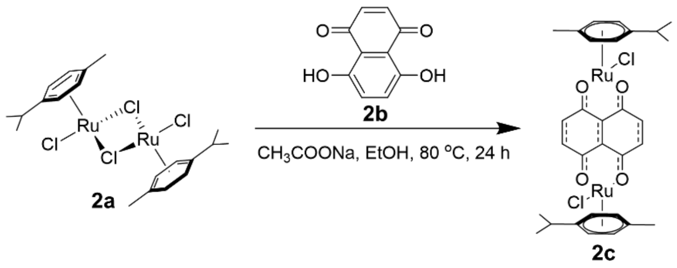

*Synthesis of compound **2c**[3]:*

2a (0.49 g, 0.80 mmol), sodium acetate (0.14 g,1.71 mmol) and **2b** (0.18 g, 0.95 mmol) were dissolved in ethanol under the protection of argon atmosphere. After heated at 80 °C for 24 h, the reaction was concentrated by rotary evaporation and washed with ethanol, water and acetone to obtain black solid **2c** (0.57 g, 95%). ^{1}H NMR (400 MHz, $CDCl_3$) δ: 6.99 (s, 4H), 5.53 (d, J = 5.7 Hz, 4H), 5.27 (d, J = 5.6 Hz, 4H), 2.95 - 2.88 (m, 2H), 2.26 (s, 6H), 1.35 (d, J = 6.9 Hz, 12H). ^{13}C NMR (100 MHz, $CDCl_3$) δ: 171.00, 137.06, 111.97, 100.38, 97.99, 82.88, 79.67, 30.78, 22.38, 17.93.

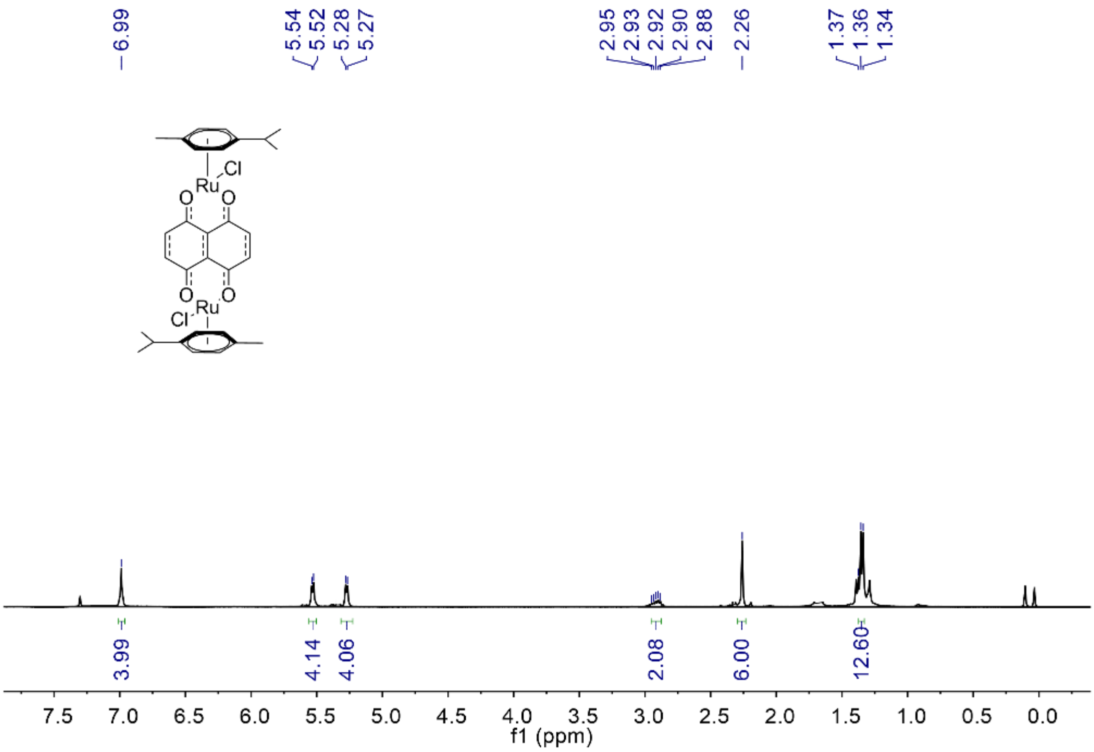

Supplementary Figure 62. ^{1}H NMR spectrum of **2c.**

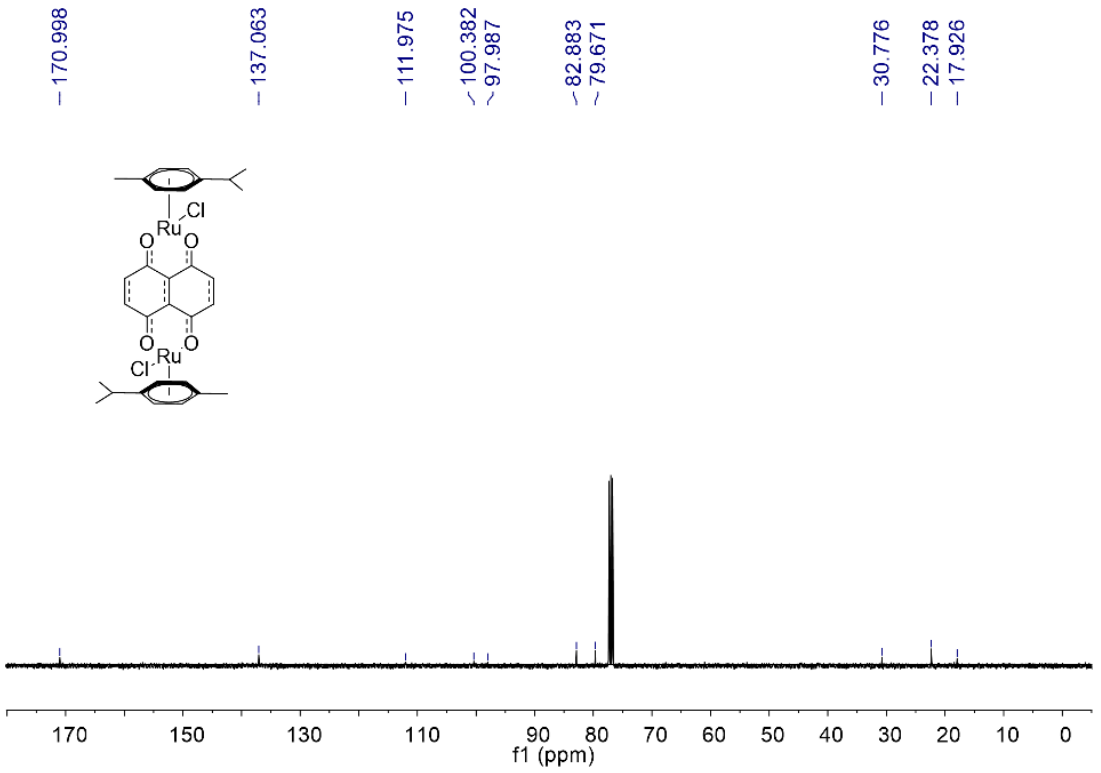

Supplementary Figure 63. ^{13}C NMR spectrum of **2c.**

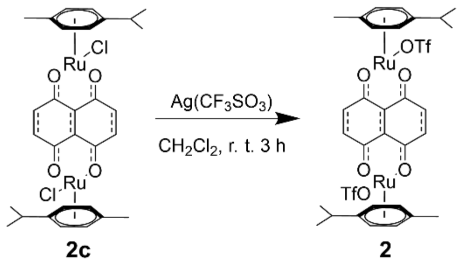

*Synthesis of compound **2**[3]:*

2c (0.57 g, 0.78 mmol) and silver trifluoromethanesulfonate ($Ag(CF_3SO_3)$) (0.41 g, 1.60 mmol) were dissolved in DCM. After stirring at room temperature for 3 h, the reaction was filtered and washed three times with methanol to obtain product **2** (0.77 g, 93%). ^1H NMR (400 MHz, CD_3OD) δ: 7.24 (s, 4H), 5.78 (d, J = 6.2 Hz, 4H), 5.53 (d, J = 6.2 Hz, 4H), 2.80 (t, J = 6.9 Hz, 3H), 2.17 (s, 6H), 1.29 (d, J = 6.9 Hz, 12H). ^{13}C NMR (100 MHz, CD_3OD) δ: 172.27, 138.09, 122.81, 119.65, 111.17, 101.71, 99.26, 82.23, 79.70, 31.63, 21.86, 17.08.

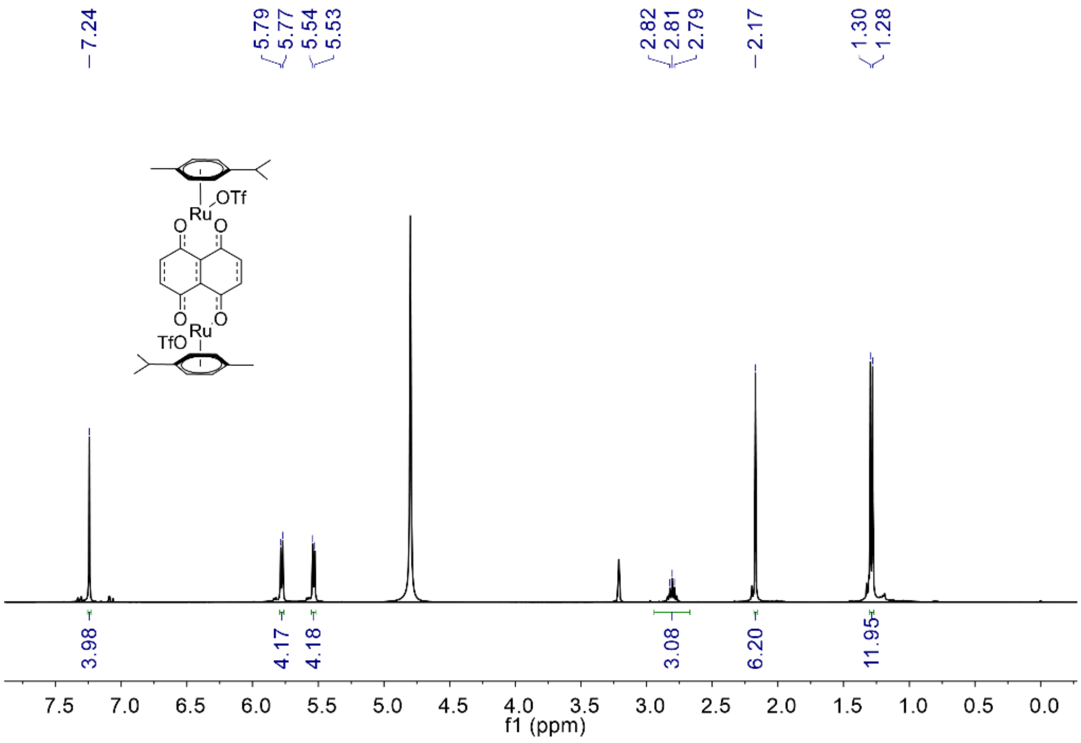

Supplementary Figure 64. ^{1}H NMR spectrum of **2.**

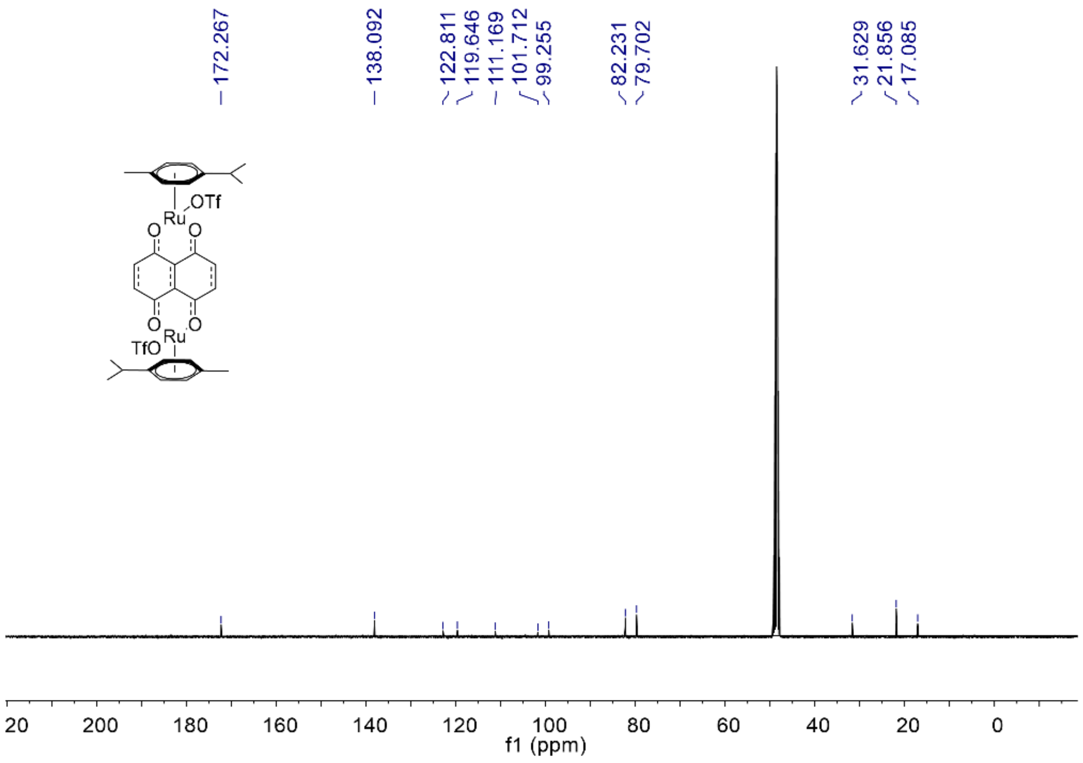

Supplementary Figure 65. ^{13}C NMR spectrum of **2.**

5. Supplementary References

1. Bai, L. Sun, P. Liu, Y. Zhang, H. Hu, W. Zhang, W. Liu, Z. Fan, Q. Lia, L. & Huang, W. Novel aza-bodipy based small molecular NIR-II fluorophores for in vivo imaging. *Chem. Commun.* **55**, 10920-10923 (2019).

2. Sahu, D. Padhy, H. Patra, D. Kekuda, D. Chu, C.-W. Chiang, I.-H. & Lin, H.-C. Synthesis and application of H-Bonded cross-linking polymers containing a conjugated pyridyl H-Acceptor side-chain polymer and various carbazole-based H-Donor dyes bearing symmetrical cyanoacrylic acids for organic solar cells. *Polymer* **51**, 6182-6192 (2010).

3. Zhao, Y. Zhang, L. Li, X. Shi, Y. Ding, R. Teng, M. Zhang, P. Cao, C. & Stang, P. J. Self-assembled ruthenium (II) metallacycles and metallacages with imidazole-based ligands and their in vitro anticancer activity. *Proc. Natl. Acad. Sci. USA* **116**, 4090–4098 (2019).
