# Supplementary material for: Construction of emissive ruthenium(II) metallacycle over 1000 nm wavelength for in vivo biomedical applications
Source: Nat Commun. 2022 Apr 14;13:2009. doi: 10.1038/s41467-022-29572-2 (PMC9010459; doi:10.1038/s41467-022-29572-2)
Supplement: Supplementary file 3 — Reporting Summary [file 41467_2022_29572_MOESM3_ESM.pdf]

Corresponding author(s): Prof. Xiaopeng Li
Last updated by author(s): 22/02/22

Reporting Summary

Nature Portfolio wishes to improve the reproducibility of the work that we publish. This form provides structure for consistency and transparency in reporting. For further information on Nature Portfolio policies, see our Editorial Policies and the Editorial Policy Checklist.

Statistics

For all statistical analyses, confirm that the following items are present in the figure legend, table legend, main text, or Methods section.

n/a	Confirmed	
☐	☒	The exact sample size (*n*) for each experimental group/condition, given as a discrete number and unit of measurement
☐	☒	A statement on whether measurements were taken from distinct samples or whether the same sample was measured repeatedly
☐	☒	The statistical test(s) used AND whether they are one- or two-sided<br>*Only common tests should be described solely by name; describe more complex techniques in the Methods section.*
☒	☐	A description of all covariates tested
☒	☐	A description of any assumptions or corrections, such as tests of normality and adjustment for multiple comparisons
☐	☒	A full description of the statistical parameters including central tendency (e.g. means) or other basic estimates (e.g. regression coefficient) AND variation (e.g. standard deviation) or associated estimates of uncertainty (e.g. confidence intervals)
☐	☒	For null hypothesis testing, the test statistic (e.g. *F*, *t*, *r*) with confidence intervals, effect sizes, degrees of freedom and *P* value noted<br>*Give P values as exact values whenever suitable.*
☒	☐	For Bayesian analysis, information on the choice of priors and Markov chain Monte Carlo settings
☒	☐	For hierarchical and complex designs, identification of the appropriate level for tests and full reporting of outcomes
☐	☒	Estimates of effect sizes (e.g. Cohen's *d*, Pearson's *r*), indicating how they were calculated

Our web collection on statistics for biologists contains articles on many of the points above.

Software and code

Policy information about availability of computer code

Data collection	NiropticsSwirViewer (version 2.0) was used to acquire NIR-II fluorescence images. ICP-MS MassHunter was used to determine of elements. FOTRIC LinkIR (version V1.1.7.50) was used to capture thermal images. Gen5 (version CHS 2.09) was used to measure the optical density. CytExpert (version 2.4) was used to collect flow cytometry data.
Data analysis	FOTRIC AnalyzIR (version 4.1.7.6300) was used to analyze the thermal images. ImageJ2/FIJI was used to contrast and overlay fluorescence images as described in the Methods section. Half maximal inhibitory concentration (IC50) was analyzed by GraphPad Prism (version 7.0). CytExpert (version 2.4) was used to analyze flow cytometry data.

For manuscripts utilizing custom algorithms or software that are central to the research but not yet described in published literature, software must be made available to editors and reviewers. We strongly encourage code deposition in a community repository (e.g. GitHub). See the Nature Portfolio guidelines for submitting code & software for further information.

Data

Policy information about availability of data

All manuscripts must include a data availability statement. This statement should provide the following information, where applicable:

- Accession codes, unique identifiers, or web links for publicly available datasets
- A description of any restrictions on data availability
- For clinical datasets or third party data, please ensure that the statement adheres to our policy

All raw and processed data will be made available upon request.

Field-specific reporting

Please select the one below that is the best fit for your research. If you are not sure, read the appropriate sections before making your selection.

☒ Life sciences ☐ Behavioural & social sciences ☐ Ecological, evolutionary & environmental sciences

For a reference copy of the document with all sections, see nature.com/documents/nr-reporting-summary-flat.pdf

Life sciences study design

All studies must disclose on these points even when the disclosure is negative.

Sample size	For cell cytotoxicity tests, n=3 was chosen as the minimal replicate number; for cellular uptake and localization by ICP-MS and the activity tests of caspase 1 and caspase 3/7, n=3 was chosen as the minimal replicate number; for in vivo pharmacokinetics, fluorescence imaging and blood chemistry tests, n=3 was chosen as the replicate number; for in vivo antitumor experiments, n=5 was chosen as the replicate number.
Data exclusions	Data were not excluded from analysis.
Replication	All replication attempts were successful.
Randomization	Cells used for imaging and mice models for different treatments were selected randomly.
Blinding	Investigators were blinded with the different treatments of mice to collect therapy data including tumor volume and body weight.

Reporting for specific materials, systems and methods

We require information from authors about some types of materials, experimental systems and methods used in many studies. Here, indicate whether each material, system or method listed is relevant to your study. If you are not sure if a list item applies to your research, read the appropriate section before selecting a response.

Materials & experimental systems

n/a	Involved in the study	
☒	☐	Antibodies
☐	☒	Eukaryotic cell lines
☒	☐	Palaeontology and archaeology
☐	☒	Animals and other organisms
☒	☐	Human research participants
☒	☐	Clinical data
☒	☐	Dual use research of concern

Methods

n/a	Involved in the study	
☒	☐	ChIP-seq
☐	☒	Flow cytometry
☒	☐	MRI-based neuroimaging

Eukaryotic cell lines

Policy information about cell lines

Cell line source(s)	A549 cells (human, ATCC CCL-185)<br>Hela cells (human, ATCC CCL-2)<br>HepG2 cells (human, ATCC HB-8065)<br>16HBE cells (human, ATCC CRL-2741)<br>A549cisR cells (human, Shanghai Fuheng Biological Technology)
Authentication	A549 and 16HBE cells were authenticated by short tandem repeat (STR) profiling analysis. Other cell lines have not been authenticated.
Mycoplasma contamination	Cell lines were not tested for mycoplasma contamination but no indication of contamination was observed.
Commonly misidentified lines (See ICLAC register)	No commonly misidentified cell lines were used.

Animals and other organisms

Policy information about studies involving animals; ARRIVE guidelines recommended for reporting animal research

Laboratory animals	Balb/c nude mice (female) age 5-weeks were used for building tumor models.

Wild animals	No wild animals were used in this study.
Field-collected samples	No field-collected samples were used in this study.
Ethics oversight	Mouse experiments were performed under the study protocol CCNU-IACUC-2011-058, as approved by the the Office of Scientific Research Management of Central China Normal University.

Note that full information on the approval of the study protocol must also be provided in the manuscript.

Flow Cytometry

Plots

Confirm that:

- [x] The axis labels state the marker and fluorochrome used (e.g. CD4-FITC).
- [x] The axis scales are clearly visible. Include numbers along axes only for bottom left plot of group (a 'group' is an analysis of identical markers).
- [x] All plots are contour plots with outliers or pseudocolor plots.
- [x] A numerical value for number of cells or percentage (with statistics) is provided.

Methodology

Sample preparation	A549 cells (ATCC CCL-185) were digested by 0.25% trypsin for 2 min. After that, cells were stained with Annexin V-FITC apoptosis detection kit or PI (0.1 mg/mL) for 15 min and subsequently analyzed by flow cytometry.
Instrument	Flow cytometry was performed CytoFLEX (Beckman Coulter).
Software	CytExpert 2.4 was used to collect and analyze flow cytometry data.
Cell population abundance	The abundance of the relevant cell populations and the purity of the samples could not been determined because the used instrument was an analytical flow cytometry without sorting system.
Gating strategy	FSC/SSC gates were used to select mononuclear cells. Control staining was used to define positive/negative cell populations.

- [x] Tick this box to confirm that a figure exemplifying the gating strategy is provided in the Supplementary Information.
